# Growth, Dissolution and Segregation of Genetically Encoded RNA Droplets by Ribozyme Catalysis

**DOI:** 10.1002/anie.202519002

**Published:** 2026-01-07

**Authors:** Franziska Giessler, William Verstraeten, Tobias Abele, Stefan J. Maurer, Luca Monari, Kerstin Göpfrich

**Affiliations:** ^1^ Biophysical Engineering Group, Center for Molecular Biology of Heidelberg University (ZMBH) Heidelberg University Heidelberg Germany

**Keywords:** Active droplet, Liquid–liquid phase separation, Ribozyme catalysis, RNA nanotechnology, Synthetic cells

## Abstract

Active droplets, membraneless compartments driven by internal chemical reactions, are compelling models for protocells and synthetic life. A central challenge is to program their dynamic behaviors using heritable genetic information, which would grant them the capacity to evolve. Here, we create transiently active RNA droplets by integrating sites for ribozyme catalysis directly into the sequence of self‐assembling, four‐arm RNA nanostars. To enable perfusion and observe the resulting dynamics over time, we develop a method for trapping individual droplets in hydrogel cages by targeted in situ photopolymerization. This enables us to quantify the sequence‐programmable droplet dissolution and to control the degradation kinetics by choosing between fast (hammerhead) and slow (hairpin) ribozymes. Furthermore, we trigger the segregation of mixed droplet populations via the sequence‐specific cleavage of a chimeric linker RNA. The droplet‐encapsulated DNA templates code for the regrowth of new droplets, establishing the proof‐of‐principle for a minimal, genetically encoded cycle of dissolution and regrowth. By directly linking RNA sequence to droplet stability, composition, and life‐cycle dynamics, our work provides a robust platform for engineering evolvable materials and advancing the bottom‐up construction of synthetic cells.

## Graphical abstract

Ribozymes enable sequence‐encoded turnover of RNA droplet material, resulting in transiently active droplets that selectively segregate based on their genotype. This establishes a direct link between genetic information and droplet behavior, marking a step toward programmable, evolvable RNA‐based compartments with life‐like properties.

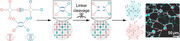

## Introduction

Active droplets are membraneless compartments with life‐like behaviors such as growth, decay, and self‐organization driven by internal chemical reactions. They could offer a route to compartmentalize early biochemistry at the origins of life^[^ ^1^ ^]^ and are compelling models for the forward‐looking goal of creating synthetic life within the fields of systems chemistry and bottom‐up synthetic biology.^[^ ^2^ ^]^ Droplets, also termed biomolecular condensates or coacervates, form spontaneously when a solution of macromolecules undergoes liquid–liquid phase separation, creating a dense phase distinct from its dilute environment driven by weak, multivalent interactions.^[^ ^3^ ^]^ Their ‘active’ nature arises from internal chemical reactions that consume energy to maintain the system out‐of‐equilibrium.^[^ ^4^ ^]^ Active droplets are of particular interest as models for minimal synthetic cells. Several studies have demonstrated that catalysis is enhanced or modulated within droplets, but often without turnover of the droplet material itself.^[^ ^5^, ^6^, ^7^, ^8^, ^9^, ^10^, ^11^ ^]^ Importantly, progress has been made in programming dynamics of the droplets themselves; for instance, fuel‐driven reaction cycles have been used to control growth and decay of peptide‐RNA droplets^[^ ^12^ ^]^ and such reaction cycles could in turn modulate DNAzyme activity.^[^ ^13^ ^]^ Theoretical work shows that active droplets should be capable of sustained growth and division under appropriate conditions.^[^ ^14^ ^]^

In these active droplets, dynamic behaviors are typically controlled by fuels and enzymes, rather than by information encoded within the droplet's own components. It would be desirable to establish a direct link between heritable genetic information and the macroscopic behavior of the compartment, a prerequisite for creating active droplets that could eventually evolve. An evolvable system, in turn, should have the capacity to adapt and show emergent behavior, a milestone towards engineering life.^[^ ^2^ ^]^ RNA is the ideal candidate to realize genetically encoded evolvable and active droplets: On the one hand, RNA can function as an information carrier and it can catalyze chemical reactions. Ribozymes, catalytically active RNA molecules, have been shown to perform ligation, cleavage, and even primitive replication and peptide bond formation.^[^ ^15^, ^16^, ^17^, ^18^, ^19^, ^20^, ^21^ ^]^ They have already been used to control the state of RNA droplets.^[^ ^11^ ^]^ It is important to note that most condensates formed from nucleic acids are driven by non‐specific electrostatic interactions between oppositely charged macromolecules, meaning there is typically little sequence specificity in these systems.^[^ ^22^, ^23^, ^24^ ^]^ On the other hand, recent advances in RNA nanotechnology allow for the design of complex RNA nanostructures.^[^ ^25^ ^]^ RNA nanotechnology has been used to create highly specific “nanostar” architectures that interact with one another based on sequence‐complementary interactions between terminal loops, the so‐called kissing loops, in absence of a polycation,^[^ ^26^, ^27^, ^28^ ^]^ hence increasing the sequence‐specificity of the liquid–liquid phase separation behavior. Such RNA droplets assemble during transcription from a DNA template and have already been equipped with aptamers for sequence‐specific recruitment of a substrate to the droplet.^[^ ^26^, ^27^ ^]^ Recently, transcription of RNA droplets has been achieved in bacterial cells^[^ ^29^ ^]^ and we have shown that RNA droplets encapsulate their own DNA template, effectively establishing a link between genotype and phenotype.^[^ ^30^ ^]^ Moreover, the dissolution of RNA droplets was achieved by strand displacement.^[^ ^28^, ^31^ ^]^

To date, however, such genetically‐encoded RNA droplets act as passive scaffolds; active droplets, with chemical turnover, have not yet been realized based on sequence‐specific interactions instead of charge multivalency. In this work, we fuse the informational and catalytic properties of RNA to create transiently active, genetically programmed droplets. We achieve this by embedding ribozyme‐mediated turnover directly into the architecture of the compartment‐forming RNA nanostars. By incorporating specific cleavage sites into the nanostar sequence design, we demonstrate programmable droplet dissolution triggered by trans‐acting ribozymes. To study individual droplets over longer periods of time and to prevent fusion events while flushing reagents, we trap RNA droplets in hydrogel cages. We show that the dissolution kinetics can be tuned by selecting different ribozymes and that we can regrow droplets from recycled DNA templates upon cleavage, effectively establishing a simple dissolution and regrowth cycle. Furthermore, by targeting a chimeric linker molecule in a mixed‐droplet population, we use sequence‐specific cleavage to induce the segregation of orthogonal droplets. These findings establish a direct, genetically encoded mechanism for controlling the stability, composition, and organization of RNA droplets, representing a step toward the engineering of responsive and evolvable materials for synthetic biology and protocell research.

## Results and Discussion

**Figure 1:**
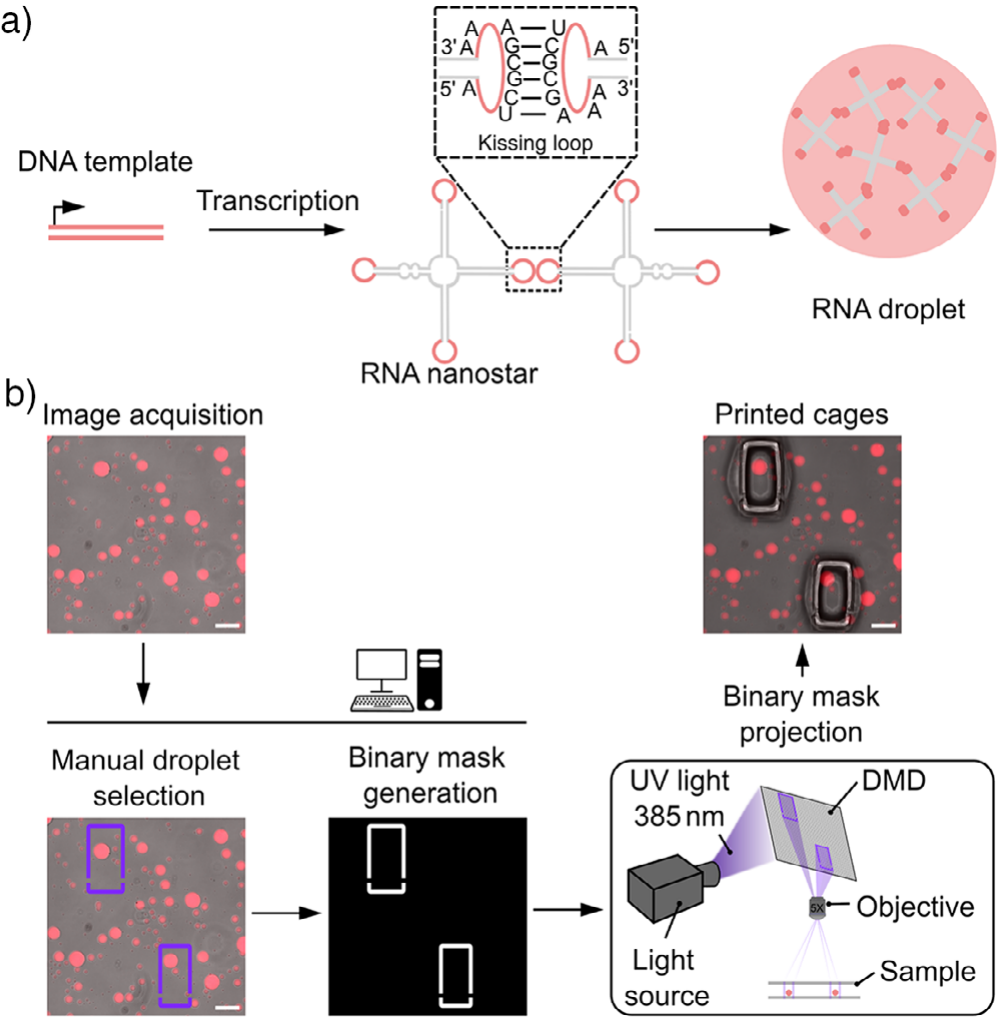
Design and in situ trapping of RNA droplets. a) Sketch of RNA droplet design and formation. A DNA template codes for a co‐transciptionally folding four‐arm RNA nanostar, which forms a droplet due to self‐complementary kissing loop interactions (dashed box).^[^ ^26^ ^]^ b) Custom‐built hardware and software environment for droplet trapping. A custom‐written software interface guides the user through image acquisition and droplet selection. A binary mask is automatically generated and projected onto the sample via a digital micromirror device (DMD) to induce hydrogel cage formation around the selected droplets by targeted photopolymerization. Fluorescent micrographs (fluorescence and brightfield overlay) of RNA droplets illustrate the experimental pipeline (droplets contain MGA and the malachite green dye, λ_ex_ = 640 nm). Scale bars: 100 µm.

To implement genetically encoded ribozyme‐regulated droplet dynamics, we built on previously established RNA droplets that form from self‐assembling four‐arm RNA nanostars.^[^ ^26^ ^]^ The RNA nanostars are transcribed from a 265 basepair long double‐stranded DNA (dsDNA) template using T7 RNA polymerase. They fold co‐transcriptionally, i.e., during transcription, into the designed monomeric four‐arm shape. Since each arm is equipped with a terminal self‐complementary kissing loop (KL), droplets form by sequence‐specific interactions between monomers (Figure 1a). The incorporation of a fluorescent light‐up aptamer (FLAP), namely a malachite green aptamer (MGA), enables fluorescence visualization of the droplet behavior. A key advantage is that transcription, folding, and droplet formation occur in a single one‐pot reaction at 37 °C. No additional steps, such as purification, component addition or temperature adjustments, are needed. Moreover, the droplet formation is sequence‐encoded in the DNA template and not triggered by electrostatic interactions between oppositely charged polymers, which is key toward our aim to link genetic information to droplet behavior.

### In Situ Trapping of RNA Droplets

In order to study droplet dynamics in a quantitative manner, we require a method to observe individual droplets over extended periods of time that allows for buffer exchange. Previously, active droplets were trapped in water‐in‐oil droplets for monitoring purposes.^[^ ^32^ ^]^ However, in this system, the surrounding oil‐phase makes perfusion steps for addition of reagents challenging to realize. In the field of microfabrication, digital micromirror devices (DMDs) have been used to create custom hydrogel microstructures by photopolymerization in order to filter and cage cells.^[^ ^33^, ^34^ ^]^

We thus repurposed a semi‐automated platform, developed in‐house,^[^ ^35^, ^36^ ^]^ for in situ trapping of individual RNA droplets inside of hydrogel chambers by targeted photopolymerization. For this purpose, we synthesized a sugar‐based photoresist consisting of the monomer glycidyl methacrylate derivatized dextran and the photoinitiator lithium phenyl‐2,4,6‐trimethylbenzoylphosphinate. We confirmed that RNA droplets are transcribed as before in the photoresist‐containing solution (Figure 1b, top left). To allow for targeted photopolymerization around individual droplets, we coupled a DMD into the beam path of a fluorescence microscope as illustrated in Figure 1b. We developed a software interface where users can select individual droplets in a live brightfield or fluorescence image with a simple mouse‐click. The rectangular cages are translated into a binary mask, which defines the illumination pattern of the DMD to locally activate the photoinitiator (for details see Supporting Information, Methods section “Targeted photopolymerization around the RNA droplets”).

We printed rectangular cages with manually adjusted dimensions around individual droplets, providing the droplets with sufficient space for growth and dissolution (Figure 1b, right). The binary masks defining the rectangular cages include small side inlets that permit reagents to flow inside and reach the trapped droplets. This design allows different reagents to be introduced sequentially into the droplet‐containing cages. The droplets remain stationary inside the cages and can be monitored for several hours, unlike droplets outside the cages which fuse and drift out of the field of view, in particular when flushing reagents (Figure S1 and Video S1).

### Ribozyme‐Based RNA Droplet Dissolution

**Figure 2:**
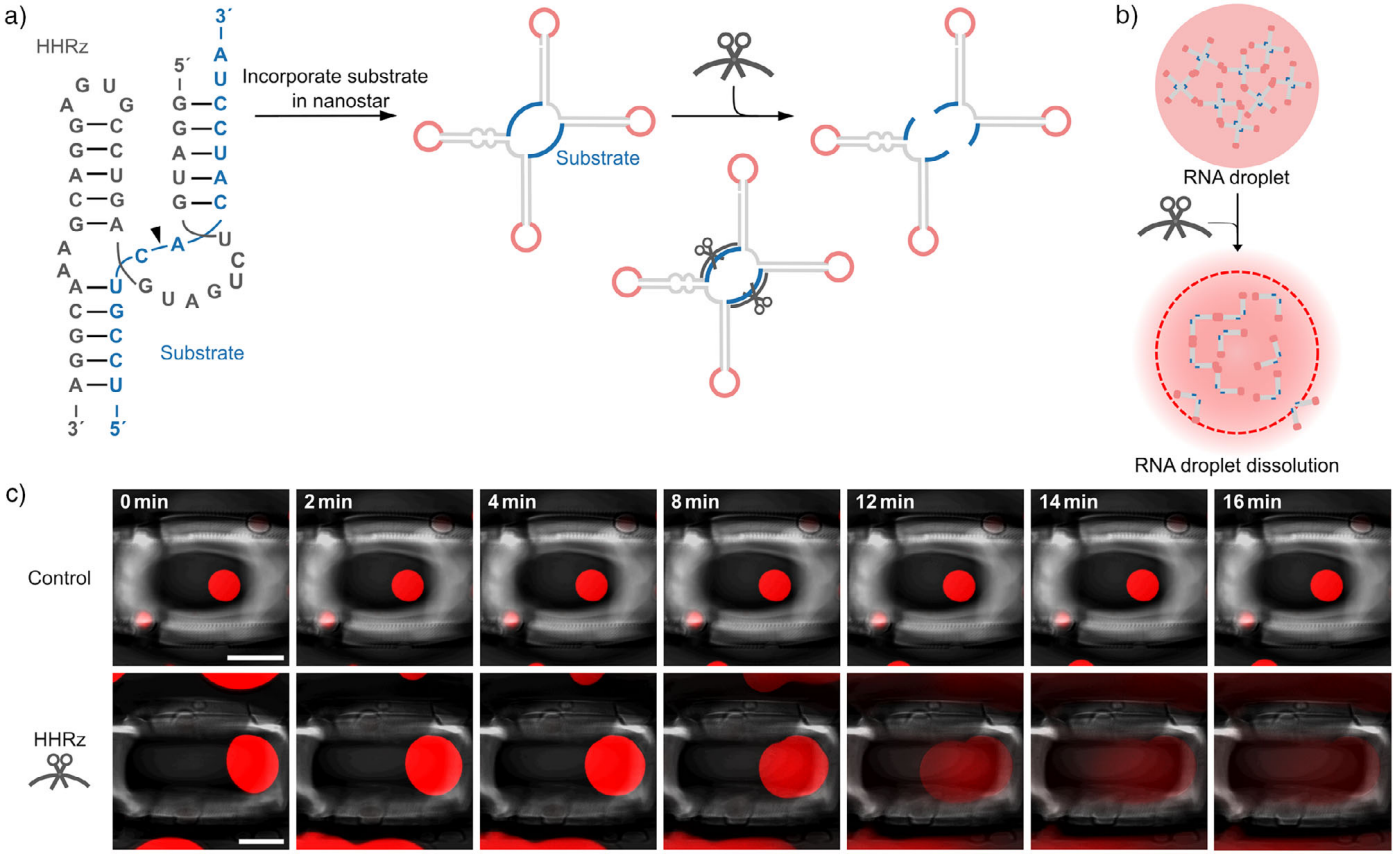
Ribozyme‐triggered RNA droplet dissolution. a) RNA sequence of the HHRz (gray) and substrate (blue). The cleavage site is indicated with a black arrow. Schematic representation illustrating the incorporation of the substrate sequence into the nanostar design. Upon addition of the ribozyme, cleavage occurs, resulting in two separate fragments, each containing two arms. b) Schematic of droplet‐forming four‐arm RNA nanostars with the incorporated ribozyme cleavage site. Cleaving the monomers into lower‐valency (two‐arm) structures upon ribozyme addition leads to droplet dissolution. c) Confocal time series of caged RNA droplets (fluorescence and brightfield overlay). Droplets contain MGA and malachite green dye, λ_ex_ = 640 nm. At t=0, IVT‐buffer (control, top row) or HHRz (bottom row) was added. Scale bars: 50 µm.

Having established a reliable setup to trap individual droplets and monitor them over time, we can observe droplet dynamics. Our goal was to introduce droplet dissolution by ribozyme activity. We thus incorporated two ribozyme cleavage sites, i.e., specific RNA sequences that are recognized by the ribozyme, into the RNA nanostar design to cleave it into two parts, each containing two arms. In particular, we chose cleavage sites for a trans‐cleaving ribozyme, which allows us to trigger cleavage upon addition of the ribozyme. Cleavage of the RNA nanostar leads to droplet dissolution because the resulting two‐arm nanostars cannot maintain the crosslinked nanostar network (Figure 2a,b).

We selected a minimal version of the hammerhead ribozyme (HHRz) (Figure 2a), with a trans‐Hoogsteen base‐pairing interaction, which has been reported to enhance catalytic activity.^[^ ^37^ ^]^ We chose this version to reduce the sequence length that needs to be incorporated into the nanostar structure, while maintaining the catalytic efficiency of the full‐length HHRz. To ensure efficient cleavage, we redesigned the RNA nanostar sequence, such that the ribozyme substrate sequence remains unpaired in the nanostar structure.

The RNA nanostar with the incorporated ribozyme target site (${\mathrm{DrA}}_{\mathrm{HHRz}}$) was transcribed at 37 °C for 24 h. The resulting droplets appeared similar to those without a target site, indicating that the incorporation of a longer single‐stranded region (15 nt) does not interfere with droplet formation (Figure 2c). We then confirmed the cleavage of ${\mathrm{DrA}}_{\mathrm{HHRz}}$ by the ribozyme via denaturing polyacrylamide gel electrophoresis (PAGE) (Figure S2).

To monitor droplet dissolution, individual droplets were first caged, and then the ribozyme was flushed into the sample. All observed droplets (*n* = 7) began to dissolve within 15 min after the addition of the ribozyme (Figure 2c, Figure S3, and Video S2). This fast dissolution is consistent with the reported cleavage kinetics of the HHRz.^[^ ^37^ ^]^ In situ transcription of the HHRz alongside already‐formed RNA droplets also resulted in rapid droplet dissolution (Figure S3b).

For the negative control, we added IVT‐buffer only (see Supporting Information, methods section “Transcription of RNA droplets”, Figure 2c). To exclude sequence‐ or binding‐dependent, non‐catalytic effects, we also tested an inactive HHRz variant (Figure S4).^[^ ^5^ ^]^ In both cases, droplets remained stable as expected. Together, these controls establish that the observed droplet disassembly is due to ribozyme catalysis.

To compare the sequence‐specific ribozyme‐based cleavage to enzyme‐mediated dissolution, we added RNase A to DrA (which lacks any engineered nuclease recognition site). RNase A led to complete dissolution within 3 min (Figure S5). RNase A non‐specifically cleaves single‐stranded RNA at C/U residues^[^ ^38^, ^39^ ^]^ distributed throughout the construct (e.g., spacers, kissing loop sequences, and the MGA). As a result, the entire RNA scaffold is degraded, and no sequence‐selective genetically encodable dissolution can be achieved in a desirable position of the nanostar.

To explore whether ribozyme catalysis can also be observed at the macroscopic scale, we transcribed the RNA droplets in a PCR tube resulting in the formation of a visible gel at the bottom. Addition of the ribozyme led to complete dissolution of the gel (Figure S6).

### Tuning Ribozyme Activity to Control Droplet Behavior

Next, we set out to alter the ribozyme kinetics. To slow down droplet dissolution to allow for more detailed monitoring, we decided to use a hairpin ribozyme (HPRz), which is reported to have a slower cleavage rate compared to the HHRz.^[^ ^40^ ^]^ The structure of the HPRz consists of four helical elements (H1 to H4) and two internal loops (A and B).^[^ ^41^ ^]^ These two independent folding domains must interact for the ribozyme–substrate complex to be catalytically active. Additionally, the HPRz exhibits biphasic kinetics, with a slow phase arising from reversible substrate binding to the inactive complex.^[^ ^42^ ^]^

**Figure 3:**
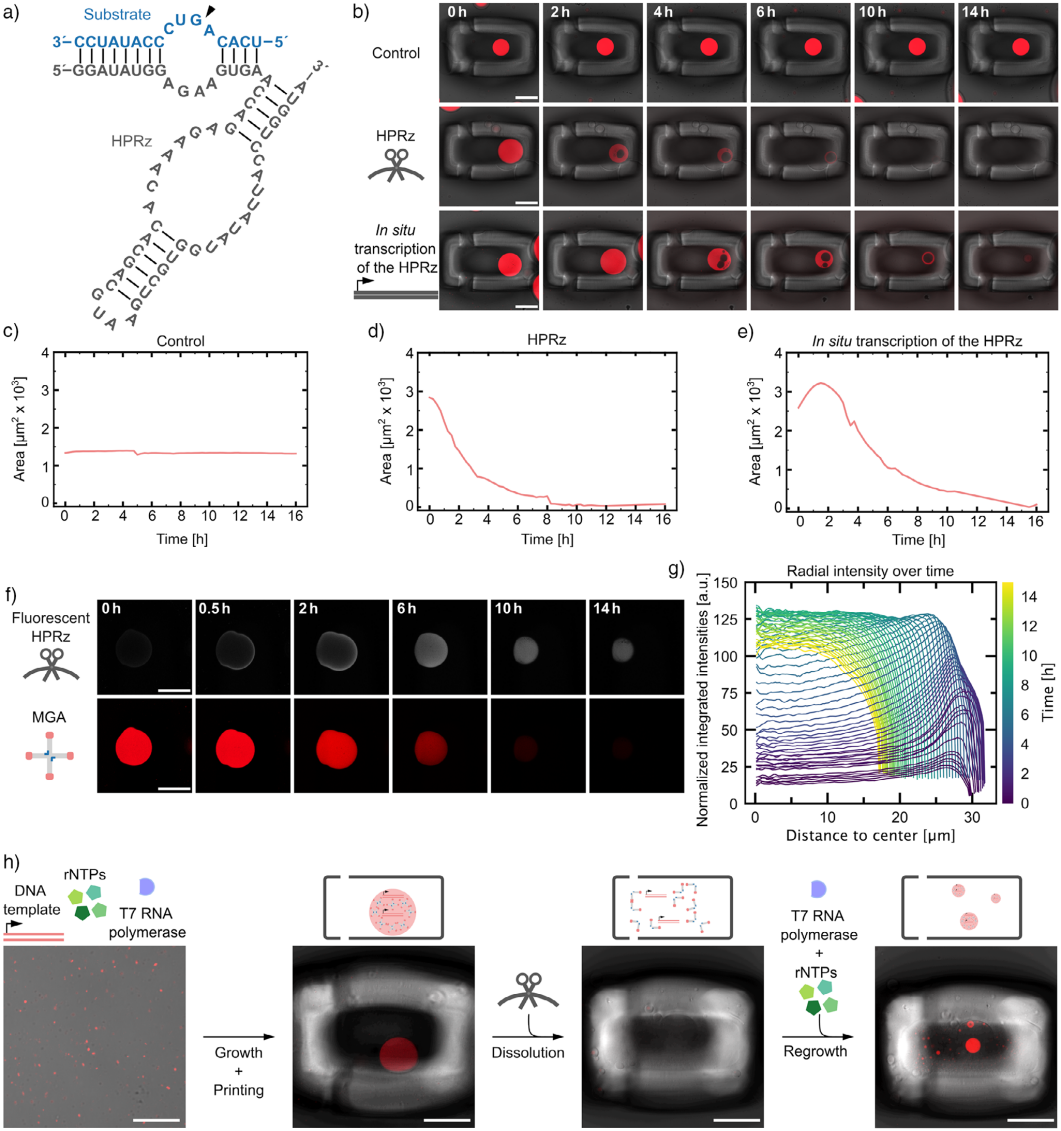
Sequence‐encoded control of ribozyme kinetics. a) RNA sequence of the HPRz (gray) and substrate (blue). The cleavage site is indicated with a black arrow. b) Confocal time series of caged RNA droplets (fluorescence and brightfield overlay). At t=0 IVT‐buffer (top row), HPRz (middle row) or in situ transcription of the HPRz (bottom row) was added. c–e) Droplet area for the droplets in b) over time. f) Confocal time series of caged RNA droplets after the addition of fluorescently labeled HPRz. At t=0, the HPRz was flushed in. The HPRz (white) is labeled with fluorescein, λ_ex_ = 488 nm. g) Radial intensity profiles showing the diffusion of fluorescently labeled HPRz into a single RNA droplet over time (see Supporting Information, methods section “Transcription of RNA droplets”). Normalized integrated intensities were measured as a function of distance from the droplet center. The color gradient represents different time points over a 14 h period. Additional replicates are shown in Figure S13. h) Schematic representation and confocal micrographs (fluorescence and brightfield overlay) showing a minimal cycle of growth, dissolution and regrowth. Key steps include transcription and droplet formation, HPRz‐induced dissolution, and droplet regrowth. For all confocal micrographs droplets (red) contain MGA and malachite green dye, λ_ex_ = 640 nm. Scale bars: 50 µm.

First, cleavage of ${\mathrm{DrA}}_{\mathrm{HPRz}}$ by the ribozyme was confirmed using PAGE (Figure S7). Again, to monitor individual droplets over time, they were trapped in a hydrogel cage, and one of three solutions was added: IVT‐buffer (control), HPRz or the IVT containing the DNA template for the HPRz, transcribing the ribozyme in situ. Upon addition of the HPRz, the droplets began to shrink gradually over several hours, occasionally accompanied by transient vacuole formation as visible in the confocal time series (Figure 3b middle, S8b middle, S9, S10, and Video S3). As expected, dissolution is considerably slower compared to cleavage with the HHRz (Figure 2). An additional negative control using an inactive version of the HPRz^[^ ^43^, ^44^ ^]^ did not show any droplet dissolution, confirming that the dissolution is again driven by sequence‐specific ribozyme cleavage (Figure S11). We also verified the sequence‐specificity of ribozyme cleavage by adding the HHRz to ${\mathrm{DrA}}_{\mathrm{HPRz}}$. As expected, the droplets remained intact (Figure S12), confirming the ribozymes' specificity for their substrate sequence rather than any single‐stranded regions. This allows for orthogonal cleavage and further demonstrates that the susceptibility to dissolution can be genetically encoded in the DNA template.

Interestingly, when transcribing the ribozyme in situ, all droplets initially grew before shrinkage set in (Figure 3b lower, S8a lower, S9, Video S4, and S5), suggesting that more RNA nanostars were also transcribed. This behavior aligns with our previous findings,^[^ ^30^ ^]^ which showed that the DNA template for the RNA nanostar is encapsulated within the droplets. Consequently, when the ribozyme is transcribed in situ, additional new RNA droplet material is transcribed along with it. Because the HPRz cleaves more slowly, this new production of RNA competes with cleavage, leading to an initial increase in droplet size before ribozyme concentration increases and thus dissolution takes over (Figures 3c–e and S8–S10).

To investigate the diffusion dynamics and mode of action of the ribozyme within RNA droplets, we labeled the ribozyme by integration of fluorescent UTPs (see Supporting Information, Methods section “Transcription of RNA droplets”) and monitored its spatial distribution using confocal microscopy (Figure 3f). Initially, the ribozyme accumulates at the droplet periphery, then gradually diffuses inward over several hours, coinciding with droplet dissolution (Figures 3f and S13). We quantified this behavior by measuring radial fluorescence intensity profiles over time. The signal initially peaks at the periphery and progressively shifts toward the center of the droplet as the droplet shrinks (Figures 3g and S13). This inward propagation suggests that cleavage activity is initially localized near the droplet surface, constrained by the internal architecture, and continues throughout the droplet as dissolution progresses.

We further tested whether RNA droplets, once dissolved, could regrow upon addition of fresh IVT‐buffer and T7 RNA polymerase, but without adding new DNA template. Indeed, new droplets formed, suggesting that the DNA still present in the sample can support further transcription and droplet formation, making growth–dissolution cycles in principle possible (Figures 3h and S14). Each droplet contains multiple copies of the DNA template, and when a large droplet dissolves, these templates are released, providing multiple nucleation points. This explains why the addition of transcription buffer results in the formation of several smaller droplets of varying sizes.

### RNA Droplet Segregation by Ribozyme Activity

Multiple RNA droplet species can be simultaneously transcribed using orthogonal KL sequences, and distinguished by confocal microscopy through the incorporation of distinct FLAPs in their nanostar designs.^[^ ^26^, ^27^ ^]^ Here we use two other nanostar designs from Fabrini et al.^[^ ^26^ ^]^ The first one, DrB was reported to form droplets orthogonal to DrA and is functionalized with the Broccoli aptamer (BrA).^[^ ^26^ ^]^ The second is a chimeric linker RNA (L) containing two KLs for DrA and two KLs for DrB. When included in a DNA template ratio of 1:2:1 (DrA: L: DrB), the system produces homogeneously mixed droplets (Figure S15).

**Figure 4:**
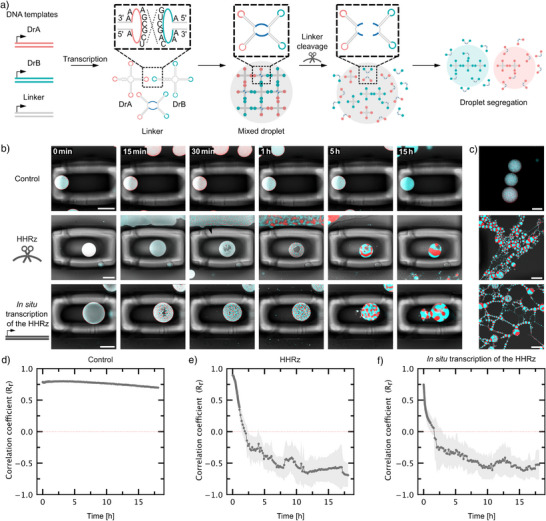
RNA droplet segregation with HHRz. a) Schematic of the cleavage strategy. Droplets are formed from two orthogonal RNA nanostars DrA and DrB with a chimeric linker (L) containing two KLs of DrA and two KLs of DrB^[^ ^26^ ^]^). We modified L with a HHRz cleavage site. Once the HHRz is added, the linker with incorporated substrate sequence is cleaved, leading to droplet segregation. b) Confocal time series of segregating RNA droplets (fluorescence and brightfield overlay). At t=0, IVT‐buffer (top row), over night transcribed ribozyme (middle row) or IVT containing the DNA template for the HHRz, transcribing the ribozyme in situ (bottom row) was added. c) Confocal micrographs of RNA droplets in uncaged regions acquired after ending the timelapse shown in (b). Red droplets contain MGA and malachite green dye, λ_ex_ = 640 nm, blue droplets contain BrA and DFHBI‐1T, λ_ex_ = 488 nm. Scale bars: 50 µm. d–f) Colocalization analysis. Pearson correlation $R_{r}$ values are plotted over time (mean ± s.d., *n* = 5 regions).

We hypothesized that we could achieve segregation of the homogeneous droplet by cleaving the linker with our previously established ribozyme cleavage strategy. We thus incorporated the substrate sequence for the highly efficient HHRz into L ($L_{\mathrm{HHRz}}$) following the same design strategy as for ${\mathrm{DrA}}_{\mathrm{HHRz}}$. The ribozyme cleavage site was positioned in such a way that the resulting fragments either contain KLs A or KLs B. Upon addition of the HHRz, the linker should be cleaved specifically, while DrA and DrB nanostars remain intact. Complete linker cleavage should disrupt DrA‐DrB interactions, leading to segregation into two distinct droplets (Figure 4a). We again confirmed cleavage of $L_{\mathrm{HHRz}}$ using PAGE (Figure S2).

DrA, $L_{\mathrm{HHRz}}$ and DrB were transcribed using a 1:2:1 DNA template ratio. We confirmed that the incorporation of the single‐stranded substrate region (15 nt) does not hinder its mixing properties (Figure 4b). As before, to monitor individual droplets over time, they were trapped in photopolymerized cages, and IVT‐buffer (control), ribozyme or the IVT containing the DNA template for the HPRz, transcribing the ribozyme in situ was added. RNA droplet segregation could be observed 15–30 min after the addition of the HHRz. Over the course of several hours the droplets first collapsed, accompanied by vacuole formation in the center of the droplet (Figure 4b and Video S6). Complete separation into two droplets was not observed for the monitored droplets, they remained connected at the interface. However, smaller droplets that were not in focus in the caged region, as well as uncaged droplets, were fully segregated, forming long chains of alternating droplet types (Figure 4c and S16). This suggests that shear forces from flow in the microfluidic chamber may facilitate segregation outside the caged regions. From experiments with the HHRz dissolving ${\mathrm{DrA}}_{\mathrm{HHRz}}$ we know that the cleavage reaction is highly efficient and occurs within minutes. Additionally, KLs A and B were designed to be orthogonal.^[^ ^26^ ^]^ We therefore attributed the incomplete segregation of the RNA droplets to the viscosity of the droplets which slows down the separation process, and to non‐specific electrostatic interactions between the monomers – although incomplete cleavage at the interface cannot be entirely excluded. To quantitatively describe the segregation kinetics, we determined the Pearson's correlation coefficient ($R_{r}$) over time as a measure of colocalization between DrA and DrB. A value of $R_{r}=1$ corresponds to perfect colocalization, whereas $R_{r}=-1$ represents perfectly anticorrelated data. Initially, $R_{r}$ decreased rapidly within the first 2 h before stabilizing around −0.5. Together, these results confirm that ribozyme‐mediated linker cleavage effectively triggers droplet segregation. We wanted to further investigate the efficiency of the ribozyme approach by comparing it to enzymatic segregation strategies using RNase H. As RNase H is known to specifically degrade the RNA in basepaired DNA‐RNA hybrids, we added RNase H together with a DNA strand complementary to the single stranded RNA region in $L_{\mathrm{HPRz}}$.^[^ ^38^, ^39^ ^]^ As expected, RNase H cleaved the linker, leading to demixing of the RNA droplets. Remarkably, we found that the catalytically active ribozyme performed just as efficiently as RNase H in cleaving the linker and disrupting droplet interactions (Figure S17). Again, these observations suggest that un‐cleaved linker is unlikely to be the only cause for incomplete segregation of the droplets. Notably, the ribozyme‐cleavage strategy thus allowed us to genetically encode the droplet's capacity to segregate, whereas RNase H would degrade any DNA‐RNA hybrid. To confirm that segregation is not due to non‐specific cleavage of the single‐stranded region of the linker, we studied the stability of the mixed droplets over time. Mixed droplets (using $L_{\mathrm{HPRz}}$) remained stable for several days, with non‐specific segregation occurring only after 6 days, likely due to degradation of the single‐stranded RNA regions in L (Figure S18). Since our experiments focus on effects within a 48h window, we conclude that spontaneous segregation is negligible in this time frame.

## Conclusion

In this study, we demonstrate that ribozyme catalysis can serve as a genetically encodable mechanism to control the stability, composition, and organization of RNA‐based droplets. To study the dissolution kinetics of RNA droplets, we repurposed an in‐house developed semi‐automated platform^[^ ^35^, ^36^ ^]^ to trap individual droplets in situ through local photopolymerization. By embedding ribozyme target sequences into self‐assembling RNA nanostars, we achieved droplet dissolution and drove the segregation of mixed droplet populations. Crucially, we were able to modulate the dissolution kinetics by selecting different ribozymes and their respective target sequences. We find that the HHRz drives rapid droplet disassembly within minutes, while the HPRz acts on timescales of hours. When transcribed in situ, the HPRz exhibits a dynamic interplay between droplet growth and degradation, suggesting the potential for designing self‐regulating systems in which assembly, disassembly, and reassembly occur in a cyclic fashion. Achieving such sustained, life‐like cycles of growth and division will require further development of mechanisms to independently tune the rates of these competing processes.

As the field of active droplets advances, we envision that the droplet trapping technology by photopolymerization will provide a powerful tool for synthetic cell research, enabling precise monitoring and manipulation of synthetic cells, with minimal perturbation for longer periods of time.

Because RNA droplets are directly transcribed from DNA templates, the information encoded in their sequence directly determines their macroscopic behavior. Due to the sequence specificity of ribozyme cleavage, only droplets containing the matching substrate are subject to dissolution, while mutated droplets should escape catalysis. Note that droplet segregation has previously been achieved for DNA droplets, triggered either by light^[^ ^39^ ^]^ or RNase H activity.^[^ ^38^, ^39^ ^]^ In these cases, however, segregation was not linked to a specific sequence. Linking DNA‐encoded information to the droplet's capacity to segregate opens a path toward genotype‐dependent selection and evolution in synthetic RNA compartments.

We further show that RNA droplets, once dissolved, can regrow without the addition of new DNA template, suggesting that the DNA released upon cleavage can be recycled for droplet formation. This establishes a minimal cycle of growth and decay, encoded entirely in nucleic acid sequences. Beyond controlling individual droplets, we demonstrate that ribozyme activity can induce transitions between mixed and demixed states. Such programmable segregation lays the foundation for engineering RNA droplets capable of asymmetric division. Just as living cells employ tightly regulated mechanisms for division, ribozyme‐mediated cleavage offers a genetically encoded strategy to split condensates into distinct, self‐contained compartments that retain the capacity for regrowth.

A defining feature of our system is that its dynamic behavior does not rely on externally supplied fuel consumption. Instead, disassembly is an intrinsic property encoded within the RNA sequence, with the energy for turnover released via ribozyme‐catalyzed phosphodiester bond cleavage. In this sense, our droplets already represent a genetically programmed form of transiently active condensates, linking heritable genetic information and catalytic activity, thus laying the foundation for evolving catalytically active droplets. At the same time the repertoire of ribozymes goes beyond phosphodiester bond cleavage as ribozymes are also capable of fuel‐consuming reactions using cofactors like S‐adenosyl methionine.^[^ ^45^ ^]^ Harnessing such reactions could couple droplet turnover to fuel consumption, providing a route toward more complex RNA‐based compartments with integrated metabolic activity. Looking further forward, the versatility of ribozymes offers numerous possibilities. Beyond cleavage, ribozymes capable of ligation or polymerization could be incorporated to support self‐sustained RNA synthesis,^[^ ^8^ ^]^ advancing toward synthetic protocells driven entirely by RNA catalysis. Future in vitro selection of ribozymes could focus on their catalytic activity within the highly charged environment of condensates, rather than in bulk. In this way, our work provides a genetically encoded route towards fuel‐dependent active droplets, closing the gap between passive RNA condensates and protocell‐like systems with evolvable, life‐like behavior.

## Conflict of Interests

S.M., T.A., and K.G. are named inventors on a patent by the Max Planck Society that covers parts of the technology described.

## Supporting information



Supporting Information

Supplemental Video 1

Supplemental Video 2

Supplemental Video 3

Supplemental Video 4

Supplemental Video 5

Supplemental Video 6

## Data Availability

The data that support the findings of this study are available in the supplementary material of this article.

## References

[1] R. R. Poudyal , F. Pir Cakmak , C. D. Keating , P. C. Bevilacqua , Biochemistry 2018, 57, 2509–2519. 29560725 10.1021/acs.biochem.8b00081 PMC7276092

[2] C. M. E. Kriebisch , O. Bantysh , L. B. Pellejero , A. Belluati , E. Bertosin , K. Dai , M. d. Roy , H. Fu , N. Galvanetto , J. M. Gibbs , S. S. Gomez , G. Granatelli , A. Griffo , M. Guix , C. O. Gurdap , J. Harth‐Kitzerow , I. S. Haugerud , G. Häfner , P. Jaiswal , S. Javed , A. Karimi , S. Kato , B. A. K. Kriebisch , S. Laha , P.‐W. Lee , W. P. Lipinski , T. Matreux , T. C. T. Michaels , E. Poppleton , A. Ruf , et al., Chem 2025, 11, 3.

[3] A. A. Hyman , C. A. Weber , F. Jülicher , Annu. Rev. Cell Dev. Biol. 2014, 30, 39–58. 25288112 10.1146/annurev-cellbio-100913-013325

[4] T. Beneyton , D. Krafft , C. Bednarz , C. Kleineberg , C. Woelfer , I. Ivanov , T. Vidaković‐Koch , K. Sundmacher , J.‐C. Baret , Nat. Commun. 2018, 9, 2391. 29921909 10.1038/s41467-018-04825-1 PMC6008305

[5] B. Drobot , J. M. Iglesias‐Artola , K. Le Vay , V. Mayr , M. Kar , M. Kreysing , H. Mutschler , T. D. Tang , Nat. Commun. 2018, 9, 3643. 30194374 10.1038/s41467-018-06072-w PMC6128941

[6] R. R. Poudyal , C. D. Keating , P. C. Bevilacqua , ACS Chem. Biol. 2019, 14, 1243–1248. 31181897 10.1021/acschembio.9b00205 PMC8819944

[7] R. R. Poudyal , R. M. Guth‐Metzler , A. J. Veenis , E. A. Frankel , C. D. Keating , P. C. Bevilacqua , Nat. Commun. 2019, 10, 490. 30700721 10.1038/s41467-019-08353-4 PMC6353945

[8] K. Le Vay , E. Y. Song , B. Ghosh , T.‐Y. D. Tang , H. Mutschler , Angew. Chem. Int. Ed. 2021, 60, 26096–26104. 10.1002/anie.202109267 PMC9299051 34569680

[9] J. M. Iglesias‐Artola , B. Drobot , M. Kar , A. W. Fritsch , H. Mutschler , T.‐Y. Dora Tang , M. Kreysing , Nat. Chem. 2022, 14, 407–416. 35165426 10.1038/s41557-022-00890-8 PMC8979813

[10] A. M. Küffner , M. Prodan , R. Zuccarini , U. Capasso Palmiero , L. Faltova , P. Arosio , ChemSystemsChem 2020, 2, e2000001.

[11] K. K. Le Vay , E. Salibi , B. Ghosh , T. D. Tang , H. Mutschler , Elife 2023, 12, e83543. 37326308 10.7554/eLife.83543 PMC10275638

[12] C. Donau , F. Späth , M. Sosson , B. A. Kriebisch , F. Schnitter , M. Tena‐Solsona , H.‐S. Kang , E. Salibi , M. Sattler , H. Mutschler , J. Boekhoven , Nat. Commun. 2020, 11, 5167. 33056997 10.1038/s41467-020-18815-9 PMC7560875

[13] A.‐L. Holtmannspötter , C. Machatzke , C. Begemann , E. Salibi , C. Donau , F. Späth , J. Boekhoven , H. Mutschler , Angew. Chem. Int. Ed. 2024, 63, e202412534. 10.1002/anie.202412534 39119638

[14] D. Zwicker , R. Seyboldt , C. A. Weber , A. A. Hyman , F. Jülicher , Nature Physics 2017, 13, 408–413.

[15] K. Kruger , P. J. Grabowski , A. J. Zaug , J. Sands , D. E. Gottschling , T. R. Cech , Cell 1982, 31, 147–157. 6297745 10.1016/0092-8674(82)90414-7

[16] C. Guerrier‐Takada , K. Gardiner , T. Marsh , N. Pace , S. Altman , Cell 1983, 35, 849–857. 6197186 10.1016/0092-8674(83)90117-4

[17] W. K. Johnston , P. J. Unrau , M. S. Lawrence , M. E. Glasner , D. P. Bartel , Science 2001, 292, 1319–1325. 11358999 10.1126/science.1060786

[18] D. P. Horning , G. F. Joyce , Proc. Natl. Acad. Sci. USA 2016, 113, 9786–9791. 27528667 10.1073/pnas.1610103113 PMC5024611

[19] B. Zhang , T. R. Cech , Nature 1997, 390, 96–100. 9363898 10.1038/36375

[20] L. Zhou , D. K. O'Flaherty , J. W. Szostak , J. Am. Chem. Soc. 2020, 142, 15961–15965. 32820909 10.1021/jacs.0c06722 PMC9594310

[21] Y. Nomura , Y. Yokobayashi , Sci. Rep. 2023, 13, 8584. 37237056 10.1038/s41598-023-35584-9 PMC10219994

[22] S. Majumder , S. Coupe , N. Fakhri , A. Jain , Nat. Commun. 2025, 16, 4258. 40335475 10.1038/s41467-025-59456-0 PMC12058984

[23] H. T. Nguyen , N. Hori , D. Thirumalai , Nat. Chem. 2022, 14, 775–785. 35501484 10.1038/s41557-022-00934-z

[24] D. Wollny , B. Vernot , J. Wang , M. Hondele , A. Safrastyan , F. Aron , J. Micheel , Z. He , A. Hyman , K. Weis , J. G. Camp , T.‐Y. D. Tang , B. Treutlein , Nat. Commun. 2022, 13, 2626. 35551426 10.1038/s41467-022-30158-1 PMC9098875

[25] L. Monari , I. Braun , E. Poppleton , K. Göpfrich , *bioRxiv* 2025. 10.1038/s41467-025-66290-x PMC12669765 41326357

[26] G. Fabrini , N. Farag , S. P. Nuccio , S. Li , J. M. Stewart , A. A. Tang , R. McCoy , R. M. Owens , P. W. Rothemund , E. Franco , M. Di Antonio , L. Di Michele , Nat. Nanotechnol. 2024, 1–9. 39080489 10.1038/s41565-024-01726-x PMC11567899

[27] J. M. Stewart , S. Li , A. A. Tang , M. A. Klocke , M. V. Gobry , G. Fabrini , L. Di Michele , P. W. Rothemund , E. Franco , Nat. Commun. 2024, 15, 6244. 39080253 10.1038/s41467-024-50003-x PMC11289419

[28] H. Udono , M. Fan , Y. Saito , H. Ohno , S.‐i. M. Nomura , Y. Shimizu , H. Saito , M. Takinoue , ACS nano 2024, 18, 15477–15486. 38831645 10.1021/acsnano.3c12161 PMC11191694

[29] B. Ng , C. Fan , M. Dordevic , A. Knirsch , L. Malouf , G. Fabrini , S. P. Nuccio , R. Rubio‐Sánchez , G. Christie , M. Takinoue , P. Cicuta , L. D. Michele , Expression of nano‐engineered RNA organelles in bacteria, 2025, https://www.biorxiv.org/content/10.1101/2025.07.08.663582v2, ISSN: 2692‐8205 Pages: 2025.07.08.663582 Section: New Results. 10.1038/s41467-026-69336-w PMC13018614 41688461

[30] W. Verstraeten , M. P. Tran , A. Taskina , P. Jaiswal , I. Haugerud , C. Helbig , M. Hamberger , E. W. Green , M. Platten , C. A. Weber , K. Göpfrich , Genetic encoding and mutagenesis of RNA droplet phenotypes, 2025, https://chemrxiv.org/engage/chemrxiv/article‐details/691623e8a10c9f5ca1314dbc.

[31] A. Tang , M. Gobry , S. Li , E. Andersen , E. Franco , Nucleic Acids Res. 2025, 53, gkaf497. 40539511 10.1093/nar/gkaf497 PMC12204698

[32] A. M. Bergmann , C. Donau , F. Späth , K. Jahnke , K. Göpfrich , J. Boekhoven , Angew. Chem. Int. Ed. 2022, 61, e202203928. 10.1002/anie.202203928 PMC9400878 35657164

[33] W. Yang , H. Yu , W. Liang , Y. Wang , L. Liu , Micromachines 2015, 6, 1903–1913.

[34] H. Zhang , M. Lu , Z. Xiong , J. Yang , M. Tan , L. Huang , X. Zhu , Z. Lu , Z. Liang , H. Liu , Lab Chip 2022, 22, 1951–1961. 35377378 10.1039/d2lc00186a

[35] K. Göpfrich , T. Abele , K. Jahnke , T. Walther , M. Wegener , T. Messer , M. Hippler , Method for non‐invasive production of defined structures inside compartements and compartment, 2023, https://patents.google.com/patent/WO2023052442A1/en.

[36] K. GÖpfrich , T. Abele , S. Maurer , Method for identifying and separating a specific component of interest from a plurality of components, and device, 2024, https://patents.google.com/patent/WO2024256656A1/en.

[37] S. M. O'Rourke , W. Estell , W. G. Scott , J. Mol. Biol. 2015, 427, 2340–2347. 25981451 10.1016/j.jmb.2015.05.005 PMC4464905

[38] Y. Sato , T. Sakamoto , M. Takinoue , Sci. Adv. 2020, 6, eaba3471. 32537507 10.1126/sciadv.aba3471 PMC7269647

[39] M. P. Tran , R. Chatterjee , Y. Dreher , J. Fichtler , K. Jahnke , L. Hilbert , V. Zaburdaev , K. Göpfrich , Small 2023, 19, 2202711. 10.1002/smll.202202711 35971190

[40] N. G. Walter , J. M. Burke , Curr. Opin. Chem. Biol. 1998, 2, 303. 9667943 10.1016/s1367-5931(98)80073-2

[41] S. E. Butcher , J. E. Heckman , J. M. Burke , J. Biol. Chem. 1995, 270, 29648–29651. 8530348 10.1074/jbc.270.50.29648

[42] J. A. Esteban , A. R. Banerjee , J. M. Burke , J. Biol. Chem. 1997, 272, 13629–13639. 9153212 10.1074/jbc.272.21.13629

[43] S. Vauléon , S. Müller , ChemBioChem. 2003, 4, 220–224. 12616637 10.1002/cbic.200390035

[44] R. Pinard , D. Lambert , N. G. Walter , J. E. Heckman , F. Major , J. M. Burke , Biochemistry 1999, 38, 16035–16039. 10587425 10.1021/bi992024s

[45] C. P. M. Scheitl , M. Ghaem Maghami , A.‐K. Lenz , C. Höbartner , Nature 2020, 587, 663. 33116304 10.1038/s41586-020-2854-z PMC7116789

