# Supplementary material for: Growth, Dissolution and Segregation of Genetically Encoded RNA Droplets by Ribozyme Catalysis
Source: Angew Chem Int Ed Engl. 2026 Jan 7;65(7):e19002. doi: 10.1002/anie.202519002 (PMC12887606; doi:10.1002/anie.202519002)
Supplement: Supplementary file 1 — Supporting Information [file ANIE-65-e19002-s005.pdf]

Supporting Information: Growth, Dissolution and Segregation of Genetically Encoded RNA Droplets by Ribozyme Catalysis

Franziska Giessler+[a], William Verstraeten+[a], Tobias Abele +[a], Stefan J. Maurer +[a], Luca Monari [a], Kerstin Göpfrich*[a]

Contents

1 Materials and Methods 3

1.1 Materials . . . 3

1.2 Methods . . . 3

2 Supplementary Tables 11

2.1 DNA Sequences (coding strand 5´-3') . . . 11

2.2 Primer Sequences . . . 14

3 Supplementary Figures 15

3.1 Figure S1: Printed cages prevent droplet fusion and movement . 15

3.2 Figure S2: Denaturing polyacrylamide gel electrophoresis of hammerhead ribozyme cleavage products . . . 16

3.3 Figure S3: Dissolution of RNA droplets induced by a trans-acting hammerhead ribozyme . . . 17

3.4 Figure S4: Addition of inactive HHRz to DrA_{HHRz}. . . . 18

3.5 Figure S5: RNase A mediated droplet dissolution. . . . 19

3.6 Figure S6: Dissolution of RNA droplets forming a gel at the bottom of a PCR tube induced by a trans-acting hammerhead ribozyme . . . 20

3.7 Figure S7: Denaturing polyacrylamide gel electrophoresis of hairpin ribozyme cleavage products . . . 21

3.8 Figure S8: Additional replicate for DrA_{HPRz} . . . 22

3.9 Figure S9: Additional replicates quantifying the effect of cleavage on droplet size for DrA_{HPRz} . . . 23

3.10 Figure S10: Quantification of vacuole-to-area ratio in DrA_{HPRz} . 24

3.11 Figure S11: Addition of inactive HPRz to DrA_{HPRz}. . . . 25

3.12 Figure S12: Ribozyme cleavage is sequence-specific . . . 26

3.13 Figure S13: Additional replicates of the diffusion of fluorescently labeled HPRz into the droplet . . . 27

3.14 Figure S14: Quantification of DrA_{HPRz} regrowth upon addition of fresh buffer and T7 RNA polymerase. 28
3.15 Figure S15: Titrating linker ratios 29
3.16 Figure S16: Additional replicates of ribozyme-induced segregation of RNA droplets 30
3.17 Figure S17: Comparing the ribozyme cleavage efficiency to enzymatic cleavage using RNase H 31
3.18 Figure S18: Mixed droplets are stable for days 33

4 Supplementary Videos 34
4.1 Video S1 34
4.2 Video S2 34
4.3 Video S3 34
4.4 Video S4 34
4.5 Video S5 34
4.6 Video S6 35

5 Supplementary data 37

1 Materials and Methods

1.1 Materials

Hepes buffer (cat #BU-106-75) and NTP Bundle (cat #NU-1014L) were purchased from Jena Bioscience, polyvinyl alcohol (PVA) (cat #814894), DL-Dithiothreitol (DTT) (cat #DO632), sucrose (cat #S0389), magnesium acetate tetrahydrate (cat #1.05819), malachite green (MG) (cat #38978), 4-(N,N-dimethylamino) pyridine (cat #107700), 3,5-difluoro-4-hydroxybenzylidene imidazolinone (DFHBI) (cat #SML2697-5MG), GelRed® (cat #SCT123) lithium phenyl-2,4,6- trimethylbenzoylphosphinate (LAP) (cat #900889), 4-(dimethylamino)pyridine (cat #107700), 3-(trimethoxysilyl)propyl methacrylate (cat #440159) and glycidyl methacrylate (cat #779342) were purchased from Sigma-Aldrich. Dextran 40 kDa (cat #7626.3) was purchased from Carl Roth. HiScribe® T7 High Yield RNA Synthesis Kit (cat #E2040S), Phusion® High-Fidelity PCR Kit (cat #E0553L), RNase H(cat #M0297L), DNase I-XT (cat #M0570S), TriDye® 1 kb Plus DNA Ladder (cat #N3270S), Low Range ssRNA Ladder (cat #N0364S), Gel Loading Dye Purple (6X) (cat #B7024S) and Deoxynucleotide (dNTP) Solution Mix (cat #N0447S) were purchased from New England Biolabs. Fluorescein-12-dUTP (cat #R0101), Novex® TBE-Urea Sample Buffer (2X) (cat #LC6876) and Novex® TBE-Urea Gels, 10% (cat #EC68752BOX) were purchased from Thermo Scientific. Midori Green Advance DNA Stain (cat #MG 04) was purchased from Nippon Genetics. Nuclease-free water (cat #11-05-01-04) was purchased from Integrated DNA Technologies. Microscopy experiments were performed in bottomless 6 channel sticky-Slides VI 0.4 (cat #80608) from ibidi mounted on a glass slide (cat #KCY5.1), 24 x 60 mm thickness 1.5 from Carl Roth. Dialysis tubing was purchased from SERVA (MWCO: 12 kDa to 14 kDa, cat #44114.02). 2-component glue "eco-sil" (cat #1300 6100) was purchased from picodent. DNA templates for RNA nanostars were synthesized as double-stranded gBlocks from Integrated DNA Technologies (IDT) and dissolved in nuclease-free water ($5\,\mathrm{ng}\,\mu\mathrm{L}^{-1}$). DNA primers and DNA templates for shorter constructs (up to 90 nucleotides, HHRz, HPRz), were ordered as single-stranded (100 nmol) DNA oligos from IDT (purification: standard desalting). PCR products were purified using the QIAquick PCR Purification Kit (cat #28104) from Qiagen.

1.2 Methods

Sequence design and folding prediction of RNA

RNA nanostar sequences for droplet A (DrA), droplet B (DrB) and the linker (L) were adapted from [1]. The starting sequence was manually modified to replace GC with GG to increase transcription efficiency [2]. The end of the sequence was adjusted accordingly. To incorporate the ribozyme substrate sequence into the nanostar while ensuring that it remained unpaired, sequences were manually modified for the hammerhead ribozyme (HHRz) by changing two nucleotides in the variable Stem I and Stem II regions, respectively. The ribozyme sequence was adjusted accordingly. The substrate of the hairpin ri-

bozyme (HPRz) was optimized using ROAD [3]. The sequence of the nanostar and conserved nucleotides in the substate sequence were constrained, while the remaining variable regions were obtained using the perl script ”batchrevolvr.pl”. The prediction of RNA secondary structure was performed using the NUPACK web app and RNAfold [4, 5]. For the coding DNA strands, a sequence containing the T7 promoter (5’-GGGCATTC<u>TAATACGACTCACTATA</u>-3’, 17 nt consensus T7 promoter underlined) was added to the beginning of the equivalent DNA sequence of the RNA nanostars and ribozymes. All DNA sequences are listed in Table S1.

PCR amplification of the DNA templates

The DNA gBlocks were dissolved in nuclease-free water at a concentration of $5\,\text{ng}\,\mu\text{L}^{-1}$. ($0.5\,\text{ng}\,\mu\text{L}^{-1}$) were PCR-amplified using the Phusion® High-Fidelity PCR Kit and corresponding forward (T7 fwd) and revers (DrA rev, DrB rev, L rev) primer, listed in Table S2, at a concentration of 0.5 µM. The annealing temperature for the primers (62 °C) was determined using the NEB Tm Calculator (version 1.16.7).

Thermal annealing steps were performed in a BioRad C1000/S1000 Thermal Cycler according to the following protocol: initial denaturation at (98 °C) for 30 s; 35 amplification cycles (denaturing at (98 °C) for 10 s, annealing at (62 °C) for 15 s, elongation at (72 °C) for 15 s); final extension at (72 °C) for 10 min; hold at (4 °C). The PCR product was then purified using the QIAquick PCR Purification Kit.

For the shorter DNA templates (HHRz and HPRz), DNA oligos were dissolved in nuclease-free water at a concentration of 10 µM. Subsequently forward and reverse strand were thermally annealed by mixing equimolar amounts of each strand, heating the sample to 85 °C for 1 min, and then cooling it down to 20 °C at a rate of $-1\,°\text{C}\,\text{min}^{-1}$. All DNA sequences were stored at −20 °C.

Agarose gel electrophoresis

The correct length of the PCR products and annealed DNA template was verified using agarose gel electrophoresis (Figure SD1). In brief, a 3% (w/v) agarose gel was prepared by dissolving agarose in 1X TAE buffer. Samples were loaded in purple (6x) loading dye and the gel was run at 90 V for 1-2 h. The gel was stained using Midori Green Advance DNA Stain and bands were visualized under UV light using a Bio-Rad ChemiDoc MP Imaging System. TriDye® 1 kb Plus DNA Ladder was used as reference.

Transcription of RNA droplets

In vitro transcription (IVT) of RNA was performed as a one-pot reaction using a home made IVT-buffer consisting of 100 mM HEPES, 20 mM $Mg(OAc)_2$, 2.5 mM rNTPS each, 1.5 mM DTT, 1.5 mM spermidine and 66.6 mMsucrose. The IVT-buffer was prepared as a 3× concentrated master mix and stored at

$-20\,°C$. For transcription reactions, the IVT-buffer was diluted to 1× final concentration using nuclease free water and supplemented with $3\,ng\,\mu L^{-1}$ DNA template. DFHBI and MG dyes were added to transcription mixtures with the corresponding fluorescent light-up aptamer (FLAP) in proportions equal to 1 µL dye (1 mM) per 20 µL mixture, yielding a final concentration of approximately 45.45 µM for each dye. Reactions were initiated by adding 4 µL T7 RNA Polymerase Mix (from the HiScribe® T7 High Yield RNA Synthesis Kit) to a 40 µL reaction followed by incubation at 37 °C.

For the transcription of fluorescently labeled HPR, 15 % of the dUTP were replaced with Fluorescein-12-dUTP yielding final concentration of 0.375 mM. This results in an average incorporation of 1-2 Fluorescein-12-dUTP molecules per RNA molecule. For the transcription of DrA:DrB:L a w/w ratio of 1:1:2 of the DNA templates was used.

Targeted photopolymerization around the RNA droplets

To prevent droplet fusion, we printed cages around individual RNA droplets by photopolymerization. For photopolymerization, we used an IVT-compatible photoresist composed of $100\,g\,L^{-1}$ glycidyl methacrylate derivatized dextran (Dex-GMA) as crosslinker and $5\,g\,L^{-1}$ lithium phenyl-2,4,6- trimethylbenzoylphosphinate (LAP) as photoinitiator. Dex-GMA and LAP were dissolved together in nuclease free water in a 2x stock concentration and then mixed with the IVT reaction solution in a 1:1 ratio, yielding an IVT solution in photoresist.

Dex-GMA was synthesized in house according to an established protocol [6]. Briefly, 10 g of 40 kDa dextran and 2 g 4-(dimethylamino)pyridine were dissolved in 60 mL DMSO before adding 8.2 mL glycidyl methacrylate. The reaction was proceeded for 48 h under stirring at room temperature. The reaction was stopped by adding 16.5 mL of 1N HCl. For purification, the reaction mixture was precipitated in 500 mL ethanol, washed with ethanol twice and dissolved in Milli-Q water. Next, ethanol was removed by rotary evaporation and the remaining liquid was dialyzed in Milli-Q water using dialysis tubing with a molecular weight cut-off of 12 kDa to 14 kDa for 5 days. Lyophilization for 7 days yielded grams of pale brown powder with 58 % of the glucose subunits carrying a methacrylate group, as determined by NMR.

In order to physically seal the printed chamber around the droplets, the printed structure had to adhere to the surface of the glass slide used during confocal fluorescence imaging.

For this purpose, we used methacrylated glass slides. For their functionalization, 24 x 60 mm glass slides with 1.5 thickness were first sonicated in ethanol for 15 min and blow dried, right before plasma activation for 30 s at 200 W and 0.4 mbar 0_2 (PVA TePla 100, PVA TePla AG). Subsequently, the activated glass slides were bathed in 300ml of 2% v/v 3-(trimethoxysilyl)propyl methacrylate (TMSPMA) in Toluene overnight. Afterwards, they were washed in an ethanol bath, followed by a Milli-Q water bath and blow drying.

A 6 channel ibidi sticky-slide was mounted onto the methacrylated glass slide to create closed microfluidic chambers. To prevent adhesion of RNA droplets to the glass surface, 100 µL of 50 mg mL^{-1} polyvinyl alcohol (PVA, dissolved in nuclease-free water) was added to each chamber, incubated for 5 min, and blow-dried with nitrogen prior to sample loading. Samples (40 µL reaction mixture) were then introduced into the chambers. The chambers were sealed with 2-component glue and incubated at 37 °C in the dark for 24 h, unless stated otherwise.

For printing, a custom-engineered setup was used, consisting of a Polygon digital micromirror device (DMD) pattern illuminator (MIGHTEX Polygon 1000) coupled to a fluorescence microscope (Axio Observer 7, Carls Zeiss AG) and connected to a PC running custom-written software for printing. The software is based on Python 3.12.4 and the ZEISS ZEN Macro Environment. In brief, we first acquired a brightfield image of the sample using a 5× objective (Plan-Apochromat 5x/0,16 M27, Carl Zeiss AG). Next, the image was converted into a data array, and regions (individual droplets, ideally spaced apart) were manually selected in a custom-written graphical user interface (GUI) based on python 3.12.4. The GUI allows to manually place rectangles with manually adjusted dimensions and a slit for substrate exchange on a canvas, with the acquired image as a background. Each rectangle drawn on the canvas was internally represented within a binary array, where the outline of each rectangle was assigned a value of 1 and the background a value of 0. Consequently, the process of drawing rectangles inherently generated a binary mask, which was forwarded to the DMD through a custom interface written in Python. The interface was hosted on the PC connected to the microscope and allowed communication between our custom GUI, the DMD and the ZEN Macro Environment *via* Hypertext Transfer Protocol. The printing process was initiated through a custom ZEN Macro, which also coordinated the hardware configuration changes of the microscope necessary for printing. For irradiation, the built-in 385 nm LED of the pattern illuminator was activated for 1000 ms and projected onto the sample according to the binary mask set on the DMD. The wall thickness was set between 27 and 54 µm, depending on the size of the printed structure.

RNA droplet dissolution and segregation induced by ribozyme cleavage

For the ribozyme-mediated dissolution of DrA, DrA with the ribozyme target site (DrA_{HHRz}, or DrA_{HPRz} was transcribed as previously described. For the segregation of DrA and DrB by ribozyme-mediated linker-cleavage, DrA, DrB, and the linker with ribozyme target-site (L_{HHRz}, or L_{HPRz}) were transcribed together with a DNA template (w/v) ratio of 1:1:2 (0.75 ng µL^{-1} for DrA and DrB and 1.5 ng µL^{-1} for the linker).

Three samples for each experiment were prepared as a master mix and split, after the addition of T7 RNA Polymerase Mix, into three separate channels of an ibidi sticky-slide. The printing was performed as previously described, at

least 10 cages per channel were printed. Afterwards, the solidified 2-component glue was removed from the channel openings with tweezers. Ribozyme samples were added either by carefully pipetting 40 µL of sample into one side of the channel and pipetting 40 µL out the other side (for the controls and the hairpin ribozyme) or by using a microfluidic pump (LabS3 Shenchen Precision Pump, speed: 10 rpm) (for the hammerhead ribozyme). Samples included: (a) 1xIVT-buffer (control), (b) ribozyme transcribed overnight at 37 °C as previously described, and (c) IVT containing the DNA template for the HPRz, transcribing the ribozyme *in situ*, also prepared as previously described DFHBI (only for experiments including DrB) and MG were added to the samples in proportions equal to 1 µL dye (1 mM) per 20 µL mixture, yielding a final concentration of approximately 45.45 mM for each dye.

Channels were re-sealed using 2-component glue and the ibidi sticky-slide was placed on the Zeiss LSM 900 confocal microscope with an incubation chamber held at 37 °C. The samples were imaged for up to 16 h.

All steps were carried out in the dark (with an infrared lamp, Sanitas SIL06 Infrarotlampe 100W) to prevent unwanted polymerization of the crosslinker.

Analysis of ribozyme cleavage products by denaturing polyacrylamide gel electrophoresis (PAGE)

Droplets were prepared as described in the transcription protocol above, with the total reaction volume reduced to 10 µL. No MG or DFHBI dyes were added. Ribozymes (HPRz and HHRz) were transcribed separately following the same protocol in a final volume of 40 µL. All samples were incubated at 37 °C for 24 h using a Bio-Rad C1000/S1000 Thermal Cycler. After incubation, DNase I was added to each sample at a final concentration of 1 U/µL, followed by incubation at 37 °C for 30 min to remove the DNA template. Then, 1 µL of each droplet sample was diluted to a total volume of 50 µL using 1x IVT-buffer. For the cleavage assay, different volumes of transcribed ribozyme were added to the diluted sample: 0 µL (control), 1 µL (1×), 5 µL (5×), and 10 µL (10×). Additionally, for the ribozyme control, ribozymes were diluted to 1 µL (1×) and 5 µL (5×) in 50 µL 1x IVT-buffer without any droplet sample. The samples were incubated at 37 °C for 3 h

Following incubation, 4 µL of each sample was mixed with 4 µL of Novex® TBE-Urea Sample Buffer (2X), heated at 70 °C for 10 min, and immediately placed on ice for 5 min. Samples were then loaded onto a pre-cast 10 % denaturing polyacrylamide gel running at 150 V for 60 min. The gel was stained with GelRed® in 1xTBE for 15 min and bands were visualized under UV light using a Bio-Rad ChemiDoc MP Imaging System.

RNase H cleavage experiment

For the segregation of DrA and DrB by RNase H mediated linker-cleavage, DrA, DrB, and the linker with a long single-stranded region (L_{HPRz}, HPRz target-site) were transcribed together with a DNA template ratio of 1:1:2 (0.75 ng µL^{-1} for DrA and DrB and 1.5 ng µL^{-1} for the linker).

Four samples were prepared as a master mix and split, after the addition of T7 RNA Polymerase Mix, into four separate channels of an ibidi sticky-slide. Printing was performed as previously described, at least 10 cages per channel were printed. Afterwards, the solidified 2-component glue was removed from the channel openings with tweezers. Samples were added by carefully pipetting 40 µL of sample into one side of the channel and pipetting 40 µL out the other side. Samples included: (a) RNase H Reaction Buffer (1x) (buffer control), (b) RNase H Reaction Buffer (1x), RNase H (36 units) (RNase H only), (c) RNase H Reaction Buffer (1x), 25 µmol of each DNA strand (DNA only), (d) RNase H Reaction Buffer (1x), 25 µmol of each DNA strand, RNase H (36 units) (RNase H + DNA). DFHBI and MG were added to the samples in proportions equal to 1 µL dye (1 mM) per 20 µL mixture, yielding a final concentration of approximately 45.45 mM for each dye.

Channels were sealed using 2-component glue and the ibidi sticky-slide was placed on the confocal microscope with an incubation chamber held at 37 °C. The samples were imaged for up to 16 h.

Confocal fluorescence microscopy

A confocal laser scanning microscope LSM 900 (Carl Zeiss AG) was used for confocal microscopy. The pinhole aperture was consistently set to one Airy Unit and the experiments were performed at 37 °C. The imaging chamber was heated to a stable 37 °C prior to imaging. The images were acquired using a 20× (Plan-Apochromat 20×/0.8 Air M27, Carl Zeiss AG) or a 5× (Plan-NEOFLUAR 5x/0.15 NA, Carl Zeiss AG) objective. The 488 nm laser was used for DFHBI/broccoli aptamer (BrA) with (347 nm/503 nm) excitation/emission wavelength. The 640 nm laser was used for MG/machite green aptamer (MGA) with (628 nm/650 nm) excitations/emission wavelengths.

Image and data analysis

Images were processed using Fiji/ImageJ2 (version 2.9.0/1.53t) [7, 8]. For the confocal micrographs shown, brightness and contrast were adjusted manually and images were cropped to the size of the cage. For the droplet area analysis (Fig. 3 and Fig. S5 and S6), all images were exported as TIFF files without any applied filters. For the Pearsons correlation coefficient analysis (Fig. S4) images were exported as split-channel TIFF files to facilitate independent analysis of RNA species. A custom-written Python script was used for both droplet area (Fig. 3, Fig. S5 and Fig. S6) and Pearson correlation coefficient calculations (Fig.4, Fig. S12). Individual droplets were detected and the area and the Pearson correlation coefficient were calculated for single droplets. In cases where multiple droplets were detected within the same cage, only the largest droplet was considered for area analysis. For Pearson correlation coefficient calculations, all detected droplets were included.

Analysis of the diffusion of fluorescently labeled hairpin ribozyme into the droplets (Fig. 3 and Fig. S7) was performed using Fiji/ImageJ2 (version 2.9.0/1.53t). Image channels were split, and only the ribozyme channel labeled with fluorescein was used for further analysis. Each confocal image stack was processed with a Gaussian blur ($\sigma = 2$) to reduce high-frequency noise. To identify fluorescent regions, the image stack was duplicated and an automatic threshold was applied using the ”Default dark” method. The resulting binary image stack was converted into a mask with a dark background. The “Analyze Particles” function was then used with a minimum size threshold of 30 pixels and a circularity range of 0.80 to 1.00 to detect approximately circular fluorescent region for each image. These were displayed as overlays and stored in the ROI Manager. For each ROI, a radial intensity profile was calculated using the Radial Profile plugin, with spatial calibration enabled to report distances in micrometers. The resulting profiles were exported as comma-separated values (CSV) files for further plotting.

The Pearson correlation coefficient R_r was calculated using the formula:

$$R_r = \frac{\sum (R_i - \overline{R})(B_i - \overline{B})}{\sqrt{\sum (R_i - \overline{R})^2 \sum (B_i - \overline{B})^2}} \in [-1, 1] \tag{1}$$

where R_i or B_i is the intensity of the ith pixel in the red or blue channel respectively; $\overline{R}$ and $\overline{B}$ are the mean values of all pixel intensities of each channel.

Statistical Analysis

If not stated otherwise, all data is presented either for a single sample (whereby repeats were always conducted and the data is shown in the SI) or as mean ± standard deviation. The sample sizes (n) are stated in the figure captions.

2 Supplementary Tables

2.1 DNA Sequences (coding strand 5´-3’)

DrA:

```
  1 GGGCATTCTA ATACGACTCA CTATAGGACA GTGCTATGAG
 41 TGTGCACGGG ATCCCGACTG GCCGCATCGC GAAAGTGGCC
 81 AGGTAACGAA TGGATCCTGT GCTGCACATT AGAGTCGCTG
121 TATGACCCAT CGCGAAAGGG TCGTACAGCG GCTCTAGTGT
161 GCTGCACAGT GTCTGTGCGA CTGCACGCAT CGCGAAAGCG
201 TGTAGTCGCA TAGACATTGT GCTCACTCGT AGCATTGTCC
241 CTGTCTCCAT CGCGAAAGGA GATAG
```

$\mathrm{DrA}_{\mathrm{HHRz}}$:

```
  1 GGGCATTCTA ATACGACTCA CTATAGGACA GTGCTATGAG
 41 TGTTCCGTCA CATCCTATGC ACGGGATCCC GACTGGCCGC
 81 ATCGCGAAAG TGGCCAGGTA ACGAATGGAT CCTGTGCTGC
121 ACATTAGAGT CGCTGTATGA CCCATCGCGA AAGGGTCGTA
161 CAGCGGCTCT AGTGTGCTTC CGTCACATCC TATCGACAGT
201 GTCTGTGCGA CTGCACGCAT CGCGAAAGCG TGTAGTCGCA
241 TAGACATTGT CGTCACTCGT AGCATTGTCC CTGTCTCCAT
281 CGCGAAAGGA GATAG
```

$\mathrm{DrA}_{\mathrm{HPRz}}$:

```
  1 GGGCATTCTA ATACGACTCA CTATAGGACA GTGCTATGAG
 41 TGTTCACAGT CCCATATTCG CACGGGATCC CGACTGGCCG
 81 CATCGCGAAA GTGGCCAGGT AACGAATGGA TCCTGTGCTG
121 CACATTAGAG TCGCTGTATG ACCCATCGCG AAAGGGTCGT
161 ACAGCGGCTC TAGTGTGCTC ACAGTCCCAT ATCCTGCACA
201 GTGTCTGTGC GACTGCACGC ATCGCGAAAG CGTGTAGTCG
241 CATAGACATT GTGCTCACTC GTAGCATTGT CCCTGTCTCC
281 ATCGCGAAAG GAGATAG
```

DrB:

```
  1 GGGCATTCTA ATACGACTCA CTATAGGACA GTGCTATGAG
 41 TGTCGCGACG GAGACGGTCG GGTCCAGATA GGCCAGTCGA
 81 CAAGGTCTAT CTGTCGAGTA GAGTGTGGGC TCCGTCGCGT
121 GCACATTAGA GTCGCTGTAT GCCACAGTCG ACAAGTGGCG
161 TACAGCGGCT CTAGTGTGCT GCACAGTGTC TGTGCGACTG
201 CACCCAGTCG ACAAGGGTGT AGTCGCATAG ACATTGTGCT
241 CACTCGTAGC ATTGTCCCTG TCTCCAGTCG ACAAGGAGAT
281 AG
```

L:

```
  1 GGGCATTCTA ATACGACTCA CTATAGGACA GTGCTATGAG
 41 TGTGCACAGT GTCTGTGCGA CTGCACCCAT CGCGAAAGGG
 81 TGTAGTCGCA TAGACATTGT GCTGCACATT AGAGTCGCTG
121 TATGAGGGAT CGCGAAACCC TCGTACAGCG GCTCTAGTGT
161 GCTCGCGTGC CTCAGAGGAC CTGTCACCAG TCGACAAGGT
201 GATAGGTCCT TTGAGGTACG CGTCACTCGT AGCATTGTCC
241 CTGTCTCCAG TCGACAAGGA GATAG
```

L_{HHRz}:

```
  1 GGGCATTCTA ATACGACTCA CTATAGGACA GTGCTATGAG
 41 TGTTCCGTCA CATCCTATGC ACAGTGTCTG TGCGACTGCA
 81 CCCATCGCGA AAGGGTGTAG TCGCATAGAC ATTGTGCTGC
121 ACATTAGAGT CGCTGTATGA GGGATCGCGA AACCCTCGTA
161 CAGCGGCTCT AGTGTGCTTC CGTCACATCC TATCGCGTGC
201 CTCAGAGGAC CTGTCACGAG TCGACAACGT GATAGGTCCT
241 TTGAGGTACG CGTCACTCGT AGCATTGTCC CTGTCTCCAG
281 TCGACAAGGA GATAG
```

L_{HPRz}:

```
  1 GGGCATTCTA ATACGACTCA CTATAGGACA GTGCTATGAG
 41 TGTTCACAGT CCCATATTCT GCACAGTGTC TGTGCGACTG
 81 CACCCATCGC GAAAGGGTGT AGTCGCATAG ACATTGTGCT
121 GCACATTAGA GTCGCTGTAT GAGGGATCGC GAAACCCTCG
161 TACAGCGGCT CTAGTGTGCT TCACAGTCCC ATATCCTCGC
201 GTGCCTCAGA GGACCTGTCA CGAGTCGACA ACGTGATAGG
241 TCCTTTGAGG TACGCGTCAC TCGTAGCATT GTCCCTGTCT
281 CCAGTCGACA AGGAGATAG
```

HHRz:

```
 1 GGGCATTCTA ATACGACTCA CTATAGGATG TCTGATGAGT
41 CCGTGAGGAC GAAACGGA
```

Inactive HHRz:

```
 1 GGGCATTCTA ATACGACTCA CTATAGGATG TCTAATGAGT
41 CCGTGAGGAC AAAACGGA
```

HPRz:

```
 1 GGGCATTCTA ATACGACTCA CTATAGGATA TGGAGAAGTG
41 AACCAGAGAA ACACACGACG TAAGTCGTGG TATATTACCT
81 GGTA
```

Inactive HPRz:

```
 1 GGGCATTCTA ATACGACTCA CTATAGGATA TGGAGAAGTG
41 AACCAGAGAA AGACACGACG TAAGTCGTGG TATATTACCT
81 GGTA
```

DNA_{c1}:

```
 1 GAATATGGGA CTGTGA
```

DNA_{c2}:

```
 1 GGATATGGGA CTGTGA
```

Table S1: Sequences of the DNA templates. All sequences are written in 5'-3' direction.

2.2 Primer Sequences

T7 fwd primer:

```
1 GGGCATTCTA ATACGACTCA CTATA
```

T7 fwd primer:

```
1 GGGCATTCTA ATACGACTCA CTATA
```

DrA rev primer:

```
1 CTATCTCCTT TCGCGATGGA
```

DrB rev primer:

```
1 CTATCTCCTT GTCGACTGGA G
```

L rev primer:

```
1 CTATCTCCTT GTCGACTGGA G
```

Table S2: Sequences of the PCR primers. All sequences are written in 5'-3' direction

3 Supplementary Figures

3.1 Figure S1: Printed cages prevent droplet fusion and movement

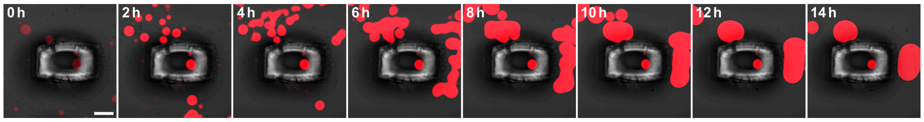

Figure S1: Printed cages prevent droplet fusion and movement. Confocal time series of caged RNA droplets (DrA_{HPRz}, contain MGA and are labeled with malachite green dye, $\lambda_{ex} = 640\,nm$), fluorescence and brightfield overlay), showing that the droplet remains inside of the cage for 14 h. The cage prevents movement and fusion with other droplets and therefore allows to observe individual droplets over time. At $t = 0$, IVT-buffer was flushed in. Scale bars: 50 µm.

3.2 Figure S2: Denaturing polyacrylamide gel electrophoresis of hammerhead ribozyme cleavage products

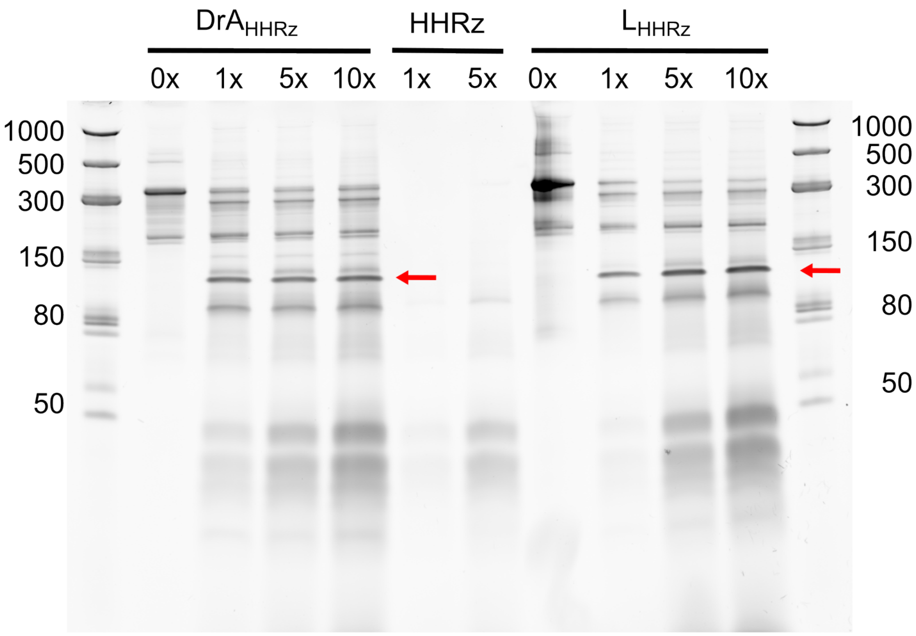

Figure S2: Denaturing polyacrylamide gel electrophoresis of hammerhead ribozyme cleavage products. *In vitro* transcribed RNA nanostars (corresponding design is written on top of the gel) were incubated with increasing volumes of ribozyme (1×, 5×, 10×), where 1× corresponds to 1 µL of ribozyme added to 1 µL of target RNA in a total volume of 50 µL. Lanes labeled 0× serve as cleavage-negative controls with no ribozyme added, the lanes labeled HHRz 1× and 5× represent ribozyme-only controls, where ribozymes were diluted (1 µL or 5 µL) in 50 µL IVT-buffer without droplet RNA (for more details see methods). Cleavage products were resolved by 10 % denaturing PAGE stained with GelRed®. Low Range ssRNA Ladder is used as reference, length in bases is reported as text close to the corresponding bands. Red arrows indicate expected cleavage products. Cleavage of full-length constructs (270 nt) is expected to yield three fragments with a lenght of 24 nt, 135 nt and 111 nt.

3.3 Figure S3: Dissolution of RNA droplets induced by a trans-acting hammerhead ribozyme

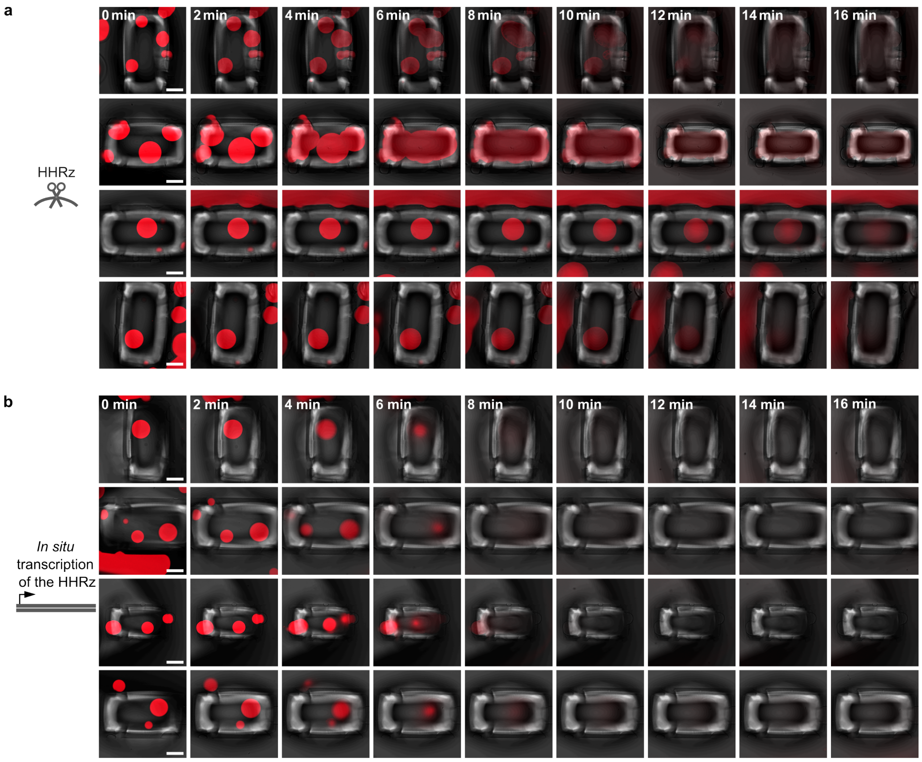

Figure S3: Dissolution of RNA droplets induced by a trans-acting hammerhead ribozyme. Confocal time series (fluorescence and brightfield overlay, droplets contain MGA and are labeled with malachite green dye, $\lambda_{ex} = 640\,\mathrm{nm}$) for multiple technical replicates (n=7, 4 are shown here) of dissolving RNA droplets using a trans-acting HHRz. Scale bars: 50 µm. At $t = 0$, the HHRz (**a**) or IVT containing the DNA template for the HHRz, transcribing the ribozyme *in situ* (**b**) was added using a microfluidic pump.

3.4 Figure S4: Addition of inactive HHRz to DrA_{HHRz}.

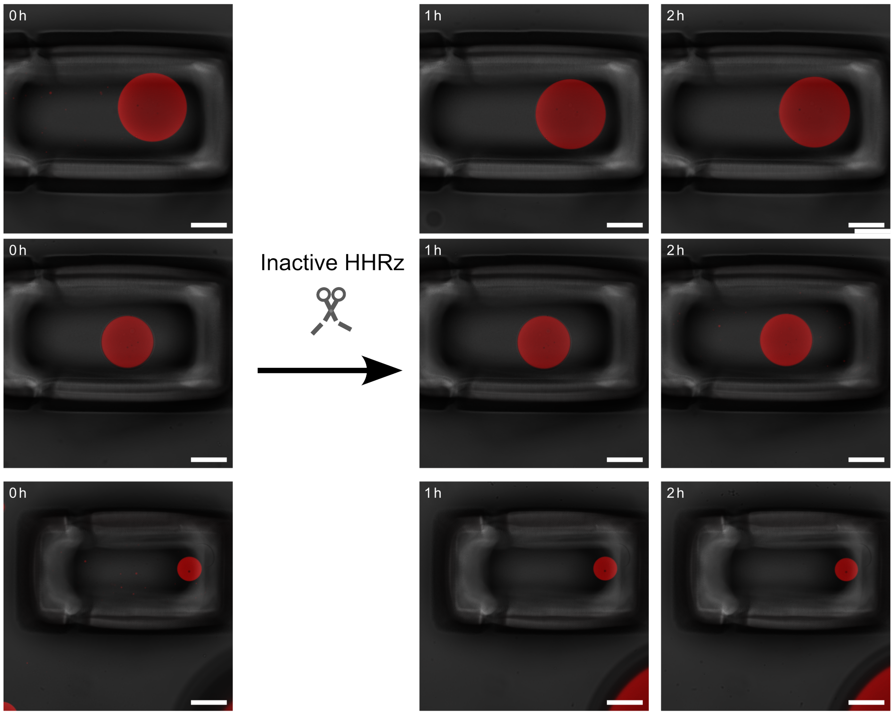

Figure S4: Confocal time series of caged RNA droplets (fluorescence and bright-field overlay). At t = 0 inactivated HHRz was added. Since HHRz cleavage happens within minutes of cleavage RNA droplets were only monitored for up to 2 hours. Droplets contain MGA and malachite green dye, $\lambda_{ex} = 640\,\text{nm}$. Scale bars: 50 µm.

3.5 Figure S5: RNase A mediated droplet dissolution.

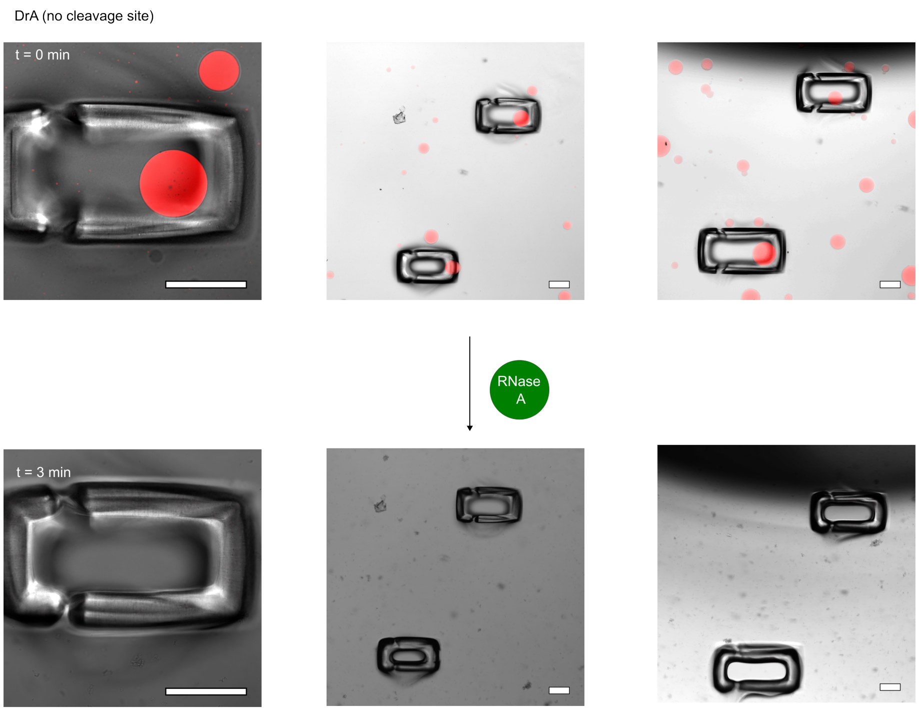

Figure S5: Dissolution of DrA by addition of RNase A. Confocal images of caged RNA droplets (fluorescence and brightfield overlay). All droplets are dissolved within 3 minutes of addition of RNaseA. Droplets contain MGA and malachite green dye, $\lambda_{ex} = 640\,\text{nm}$. Scale bars: 100 µm.

3.6 Figure S6: Dissolution of RNA droplets forming a gel at the bottom of a PCR tube induced by a trans-acting hammerhead ribozyme

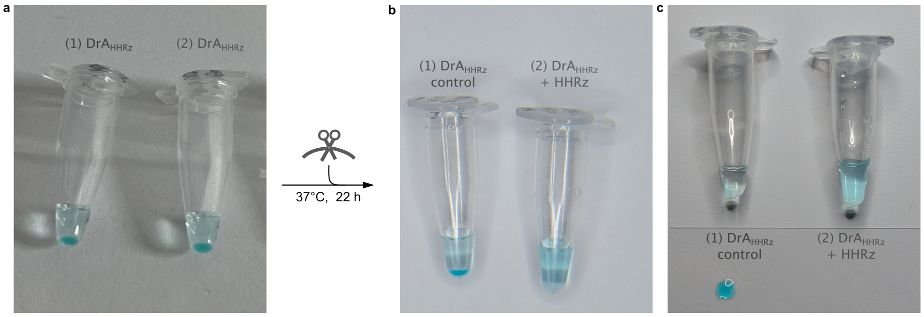

Figure S6: Dissolution of RNA droplets forming a gel at the bottom of a PCR tube induced by a trans-acting hammerhead ribozyme. **a**) RNA droplets composed of DrA_{HHRz} were formed by IVT in a 20 µL reaction at 37 °C for 24 h, resulting in a visible gel at the bottom of the PCR tube. After transcription, (1) 20 µL of IVT-buffer (control) or (2) 20 µL of separately transcribed HHRz (overnight, 37 °C) were added. Samples were mixed by pipetting, incubated for 4 h at 37 °C with shaking at 700 rpm, and then left overnight at 37 °C without shaking. **b**) PCR tubes after 22 h of incubation. The control sample (1) retains the visible RNA gel, while the HHRz-treated sample (2) **c)** In the control (1), the gel remains intact and can be extracted as a single piece with a pipette, indicating that degradation only occurs in the presence of the trans-acting HHRz.

3.7 Figure S7: Denaturing polyacrylamide gel electrophoresis of hairpin ribozyme cleavage products

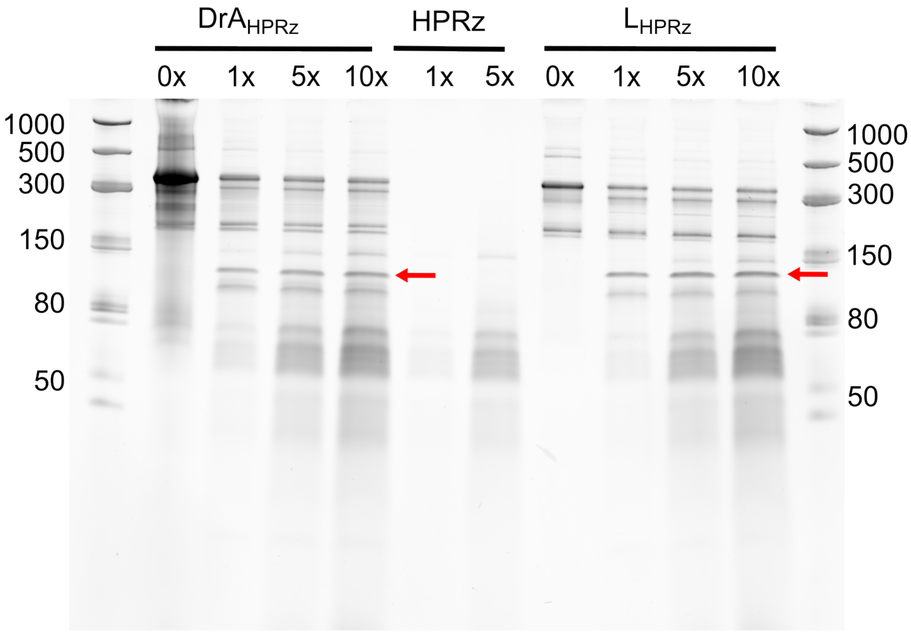

Figure S7: Denaturing polyacrylamide gel electrophoresis of hairpin ribozyme cleavage products. *In vitro* transcribed RNA nanostars (corresponding design is written on top of the gel) were incubated with increasing volumes of ribozyme (1×, 5×, 10×), where 1× corresponds to 1 µL of ribozyme added to 1 µL of target RNA in a total volume of 50 µL. Lanes labeled 0× serve as cleavage-negative controls with no ribozyme added, the lanes labeled HPRz 1× and 5× represent ribozyme-only controls, where ribozymes were diluted (1 µL or 5 µL) in 50 µL IVT-buffer without droplet RNA (for more details see methods). Cleavage products were resolved by 10 % denaturing PAGE stained with GelRed®. Low Range ssRNA Ladder is used as reference, length in bases is reported as text close to the corresponding bands. Red arrows indicate expected cleavage products. Cleavage of full-length constructs DrA_{HPRz}/ L_{HPRz} (272/274 nt) is expected to yield three fragments with a lenght of 23 nt, 135/137 nt and 114 nt.

3.8 Figure S8: Additional replicate for DrA$_{\mathbf{HPRz}}$

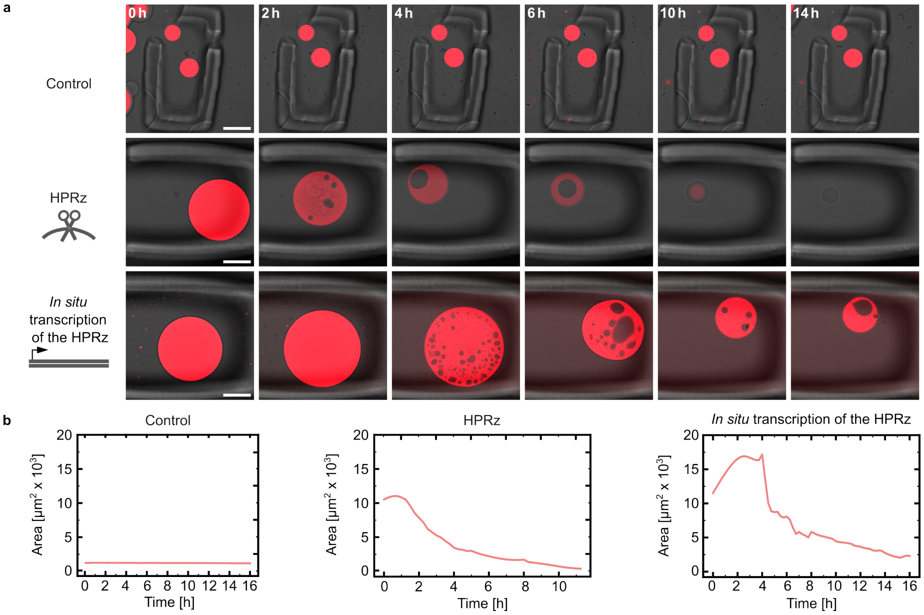

Figure S8: Additional replicates for DrA$_{\text{HPRz}}$. Confocal time series using the HPRz for dissolution of DrA$_{\text{HPRz}}$. **a)** Confocal time series (fluorescence and brightfield overlay, droplets contain MGA and are labeled with malachite green dye, $\lambda_{ex} = 640\,\text{nm}$) for a technical replicate of dissolving RNA droplets using the HPRz. At $t = 0$, buffer (top), overnight transcribed ribozyme (middle) or IVT containing the DNA template for the HPRz, transcribing the ribozyme *in situ* (bottom) was added. Scale bars: 50 µm. **b)** Droplet area over time for the droplets shown in a).

3.9 Figure S9: Additional replicates quantifying the effect of cleavage on droplet size for DrA_{HPRz}

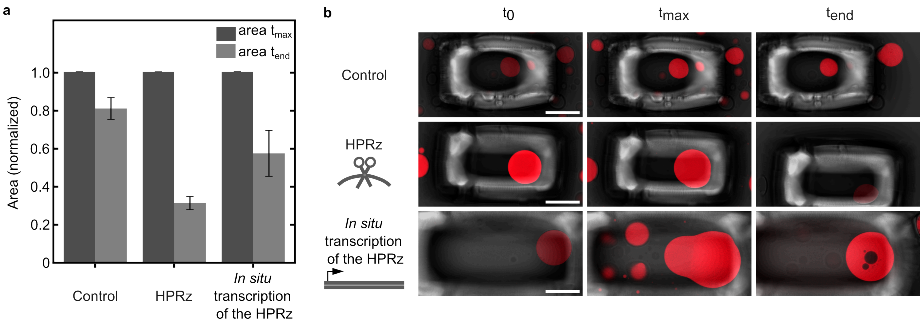

Figure S9: Effect of cleavage on droplet size for DrA_{HPRz}. HPRz was used for dissolution of DrA_{HPRz}. **a)** Comparing the normalized area of the droplets at the timepoint when they reach their maximal diameter to the droplet area at the end of the timelapse (n=6 regions, error bars represent the standard deviation). Data for the plot was pooled from one replicate. The droplet detection and area measurements were performed using a custom-written python script. **b)** Confocal micrographs (fluorescence and brightfield overlay, droplets contain MGA and are labeled with malachite green dye, $\lambda_{ex} = 640\,\text{nm}$) for one biological replicate of dissolving RNA droplets using the HPRz. One of the regions (per condition) plotted in a) is shown at the start of the timelapse (t_0), at the time where they reach their maximal size (t_{max}) and at the end of the timelapse (t_{end}), ending 22 h and 15 min after the addition of the HPRz. Scale bars: 50 µm.

3.10 Figure S10: Quantification of vacuole-to-area ratio in DrA_{HPRz}

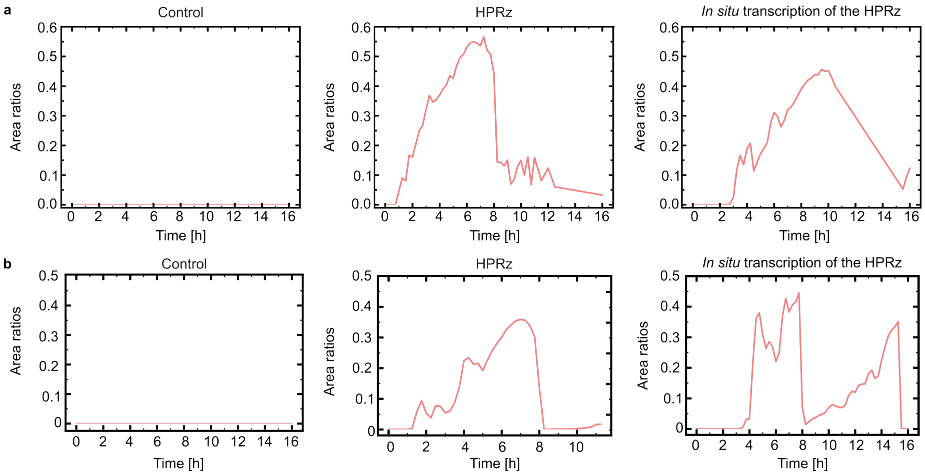

Figure S10: Quantification of vacuole-to-area ratio in DrA_{HPRz}. Transient vacuole formation observed and quantified for **a)** the droplets in Figure 3b and **b)** for the droplets in Figure S8. These vacuoles form and disappear over the observation time of 16 h. The droplet detection and vacuole-to-droplet area measurements were performed using a custom-written python script.

3.11 Figure S11: Addition of inactive HPRz to DrA_{HPRz}.

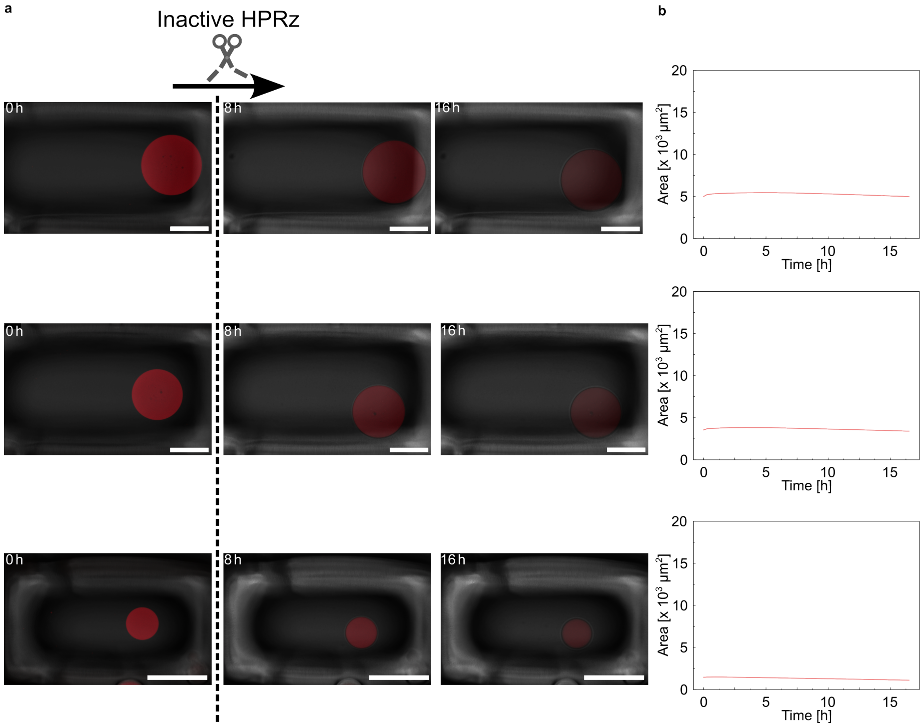

Figure S11: Addition of inactive HPRz to DrA_{HPRz}. **a)** Confocal time series of caged RNA droplets (fluorescence and brightfield overlay). At t = 0 inactivated HPRz was added. RNA droplets were only monitored for up to 16 hours after flushing. Droplets contain MGA and malachite green dye, $\lambda_{ex} = 640\,\text{nm}$. 50 µm. **b)** Quantification of the droplet area over time for individual droplets within the cages, shown in a).

3.12 Figure S12: Ribozyme cleavage is sequence-specific

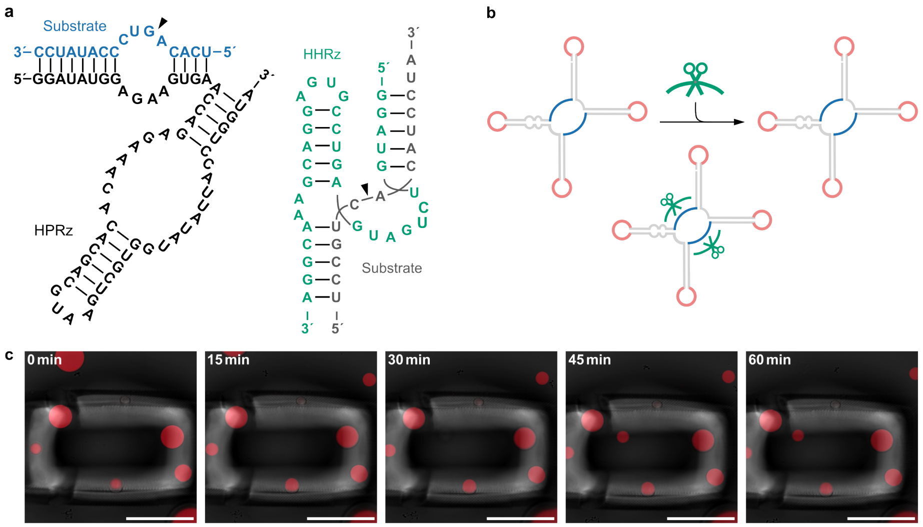

Figure S12: Ribozyme cleavage is sequence-specific. Verification of the sequence specificity of ribozyme cleavage by adding the HHRz to $\mathrm{DrA_{HPRz}}$ **a)** RNA sequence of the HPRz (black) and the HHRz (green) used in this experiment. **b)** Schematic representation illustrating the incorporation of the substrate sequence from the HPRz into the nanostar design. Addition of the HHRz ribozyme should not lead to cleavage, since the wrong substrate sequence is present. **c)** Confocal time series (fluorescence and brightfield overlay, droplets contain MGA and are labeled with malachite green dye, $\lambda_{ex} = 640$ nm) of $\mathrm{DrA_{HPRz}}$. At $t = 0$, the HHRz was added to the droplets. Scale bars: 50 µm.

3.13 Figure S13: Additional replicates of the diffusion of fluorescently labeled HPRz into the droplet

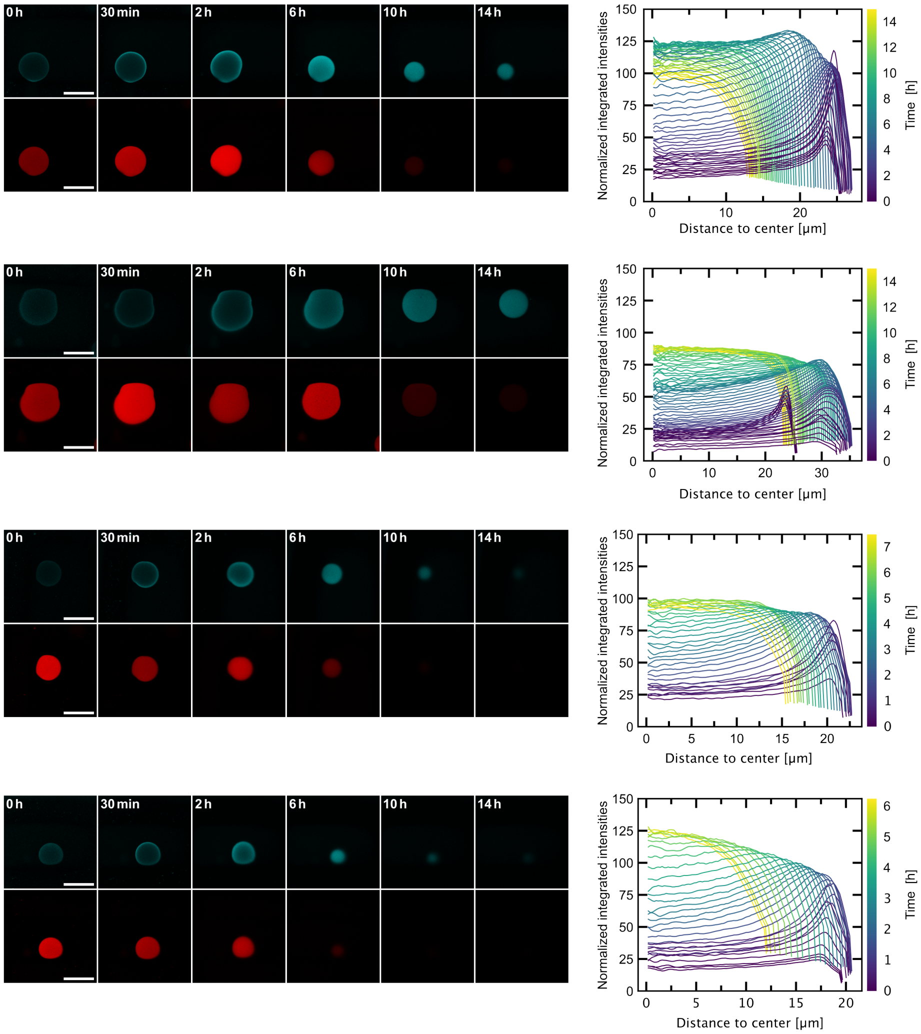

Figure S13: Confocal time series of caged RNA droplets (left) after the addition of fluorescently labeled HPRz. At $t = 0$, the HPRz was flushed in. The HPRz (blue) is labeled with fluorescein, $\lambda_{ex} = 488$ nm. RNA droplets contain MGA and are labeled with malachite green dye, $\lambda_{ex} = 640$ nm. Radial intensity profiles (right) showing the diffusion of fluorescently labeled HPRz into a single RNA droplet over time. Normalized integrated intensities were measured as a function of distance from the droplet center. The color gradient represents different time points over a 14 h period or until the droplet is dissolved. Scale bars: 50 µm.

3.14 Figure S14: Quantification of DrA_{HPRz} regrowth upon addition of fresh buffer and T7 RNA polymerase.

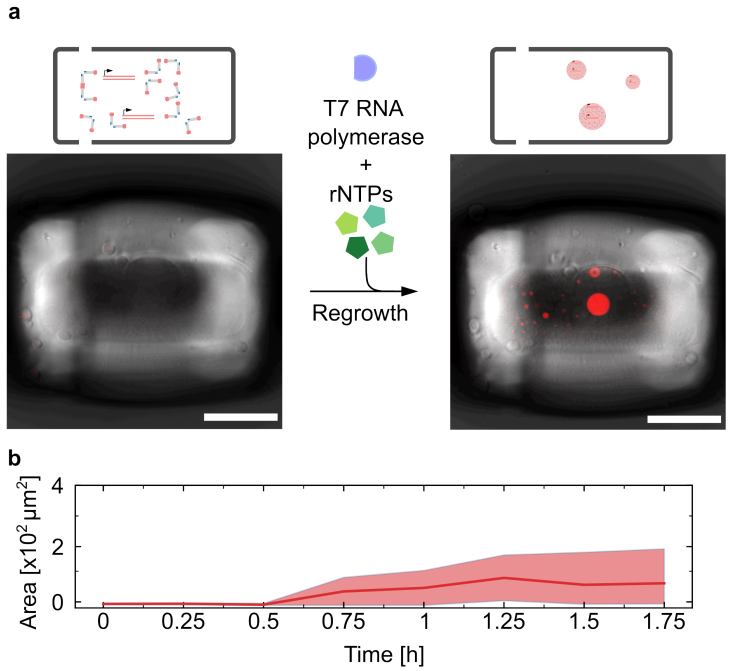

Figure S14: **a)** DrA_{HPRz} was first dissolved by addition of HPRz, after which fresh buffer and T7 polymerase were flushed. **b)** Quantification of the area of regrowing DrA_{HPRz}. The error band corresponds to the standard deviation of 2-4 droplets within one cage. Note that the number of droplets over time changes due to fusion and the formation of new droplets. Scale bars: 50 µm.

3.15 Figure S15: Titrating linker ratios

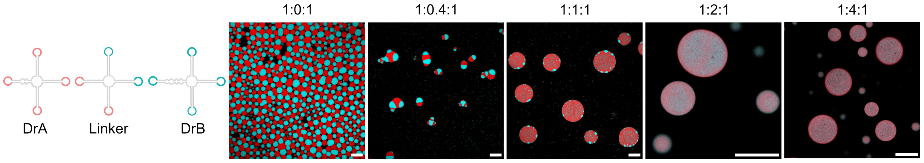

Figure S15: Titrating linker ratios. Confocal micrographs of mixed RNA droplets using different DNA template ratios for DrA: L: DrB. Red droplets contain MGA and are labeled with malachite green dye, $\lambda_{ex} = 640\,\text{nm}$, blue droplets contain BrA and are labeled with DFHBI-1T, $\lambda_{ex} = 488\,\text{nm}$. Images were acquired more than 24 h after the start of transcription. Scale bars: 50 µm.

3.16 Figure S16: Additional replicates of ribozyme-induced segregation of RNA droplets

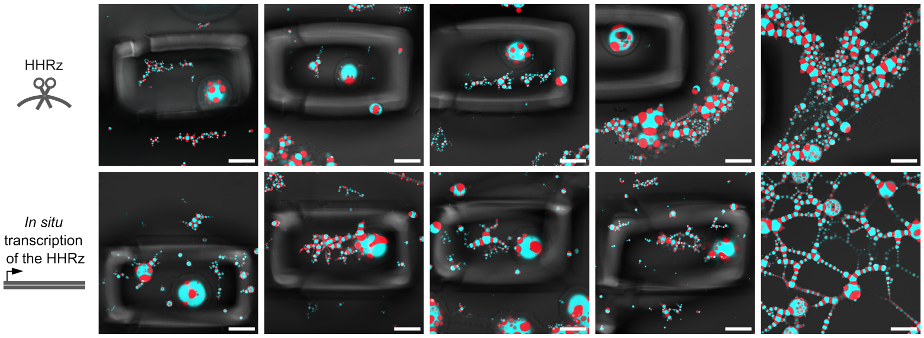

Figure S16: Confocal micrographs (fluorescence and brightfield overlay, red droplets contain MGA and are labeled with malachite green dye, $\lambda_{ex} = 640\,\text{nm}$, blue droplets contain BrA and are labeled with DFHBI-1T, $\lambda_{ex} = 488\,\text{nm}$) acquired 15 h after addition of the HHRz, as shown in Figure 4b. The focal plane was changed to show smaller droplets that were not in focus. Additionally, droplets outside of the caged regions are shown on the rightmost image for both conditions. Scale bars: 50 µm.

3.17 Figure S17: Comparing the ribozyme cleavage efficiency to enzymatic cleavage using RNase H

To compare ribozyme cleavage with enzymatic strategies, we selected the endonuclease RNase H, which specifically degrades RNA when hybridized to a complementary DNA strand in an RNA/DNA heteroduplex [9]. DrA, DrB and a linker containing two single-stranded regions (16 nt, L_{HPRz} were transcribed using a 1:1:2 DNA template ratio. Upon addition of two complementary DNA strands (DNA_{c1} and DNA_{c2}), the single-stranded regions in the linker form an RNA-DNA duplex, which are subsequently cleaved by RNase H (Figure S17a).

The segregation behavior closely resembled that observed with ribozyme cleavage: over the course of several hours the droplets first collapse, accompanied by hole formation in the center of the droplet. Subsequently, the monomers of DrA and DrB rearrange followed by their segregation. To confirm that RNase H cleavage was sequence-specific, we performed two control experiments: one without the addition of complementary DNA strands and another with complementary DNA but without RNase H. In both cases, segregation did not occur (Figure S17b).

To quantitatively describe the segregation kinetics, we also determined the Pearson's correlation coefficient (R_r) over time. Similar to the ribozyme-mediated cleavage R_r stabilizes around -0.5 , but with strong fluctuations. However, the initial phase of segregation was not captured, as the necessary steps: gluing the channels, transferring the sample to the confocal microscope, and setting up the tile regions for the timelapse took approximately 20 min. To obtain a precise t_0 corresponding to the time of addition, we would need to use the microfluidic pump in this setup as well.

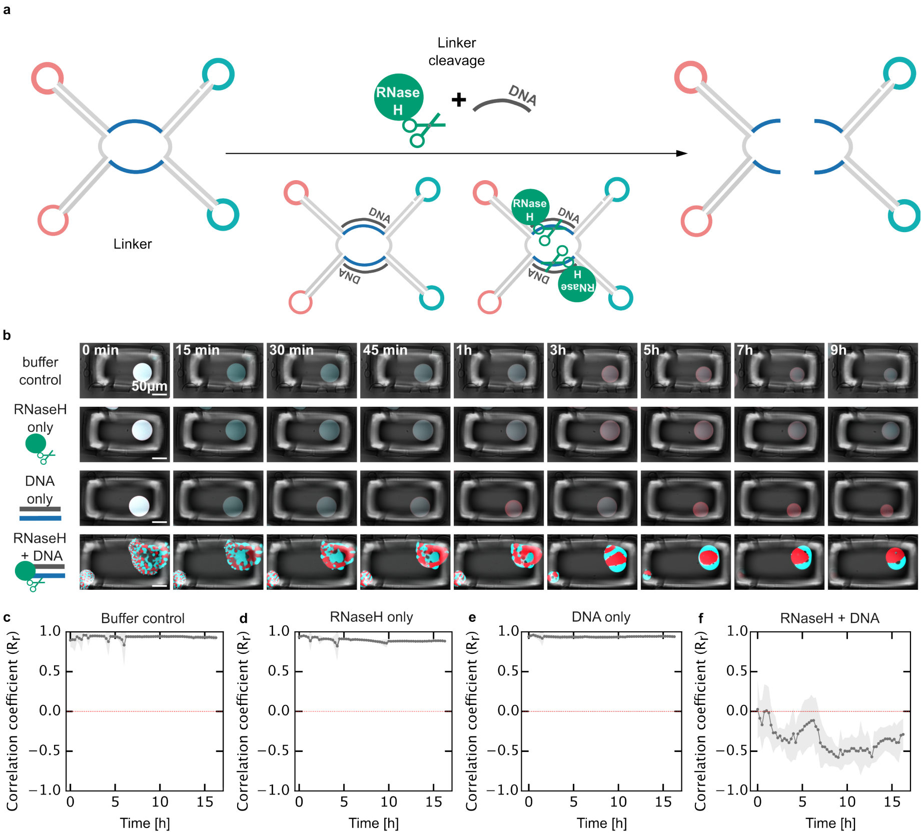

Figure S17: Comparing the ribozyme cleavage efficiency to enzymatic cleavage using RNase H. **a)** Schematic representation exemplifying linker cleavage using RNase H. Upon addition of two complementary DNA strands, the single-stranded regions in the linker form an RNA-DNA duplex, which is subsequently cleaved by RNase H. **b)** Confocal time series of caged RNA droplets (fluorescence and brightfield overlay, red droplets contain MGA and are labeled with malachite green dye, $\lambda_{ex} = 640\,\text{nm}$, blue droplets contain BrA and are labeled with DFHBI-1T, $\lambda_{ex} = 488\,\text{nm}$). $t = 0$ corresponds to the start of the timelapse about 20 min after the addition of either 1x RNase H buffer, RNase H only, DNA only or RNase H and DNA. Scale bars: 50 µm. **c)**, **d)**,**e)**, **f)** Colocalization analysis (Pearson correlation) was performed and Pearson R_r values are plotted over time (mean ± s.d., n = 3 regions for **c)**, **d)**, **f)**, n = 2 regions for **e)**).

3.18 Figure S18: Mixed droplets are stable for days

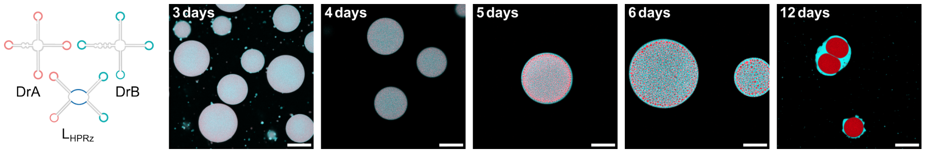

Figure S18: Mixed droplets are stable for days. Confocal micrographs of mixed RNA droplets (DrA, DrB and the respective linker L) monitoring their stability over several days. Red droplets contain MGA and are labeled with malachite green dye, $\lambda_{ex} = 640\,\text{nm}$, blue droplets contain BrA and are labeled with DFHBI-1T, $\lambda_{ex} = 488\,\text{nm}$. DrA, DrB and L_{HPRz} were transcribed together with a DNA template ratio of 1:1:2 and incubated at 37 °C. The onset of segregation without ribozyme-catalyzed linker cleavage can only be observed after 5 days and is clearly evident after 12 days. This is likely due to non-specific degradation of the single stranded regions in the linker and the BrA in DrB. Scale bars: 50 µm.

4 Supplementary Videos

4.1 Video S1

Confocal timelapse of caged RNA droplets (DrA_{HPRz}), showing that the droplet remains inside of the cage for 22 h. The cage prevents movement and fusion with other droplets and therefore allows to observe individual droplets over time. Droplets contain MGA and are labeled with malachite green dye, $\lambda_{ex} = 640\,\text{nm}$, fluorescence and brightfield overlay. From the starting point up to 22 h and 15 min, every frame is separated by a 15 min time interval. Timestamps are shown on the top left.

4.2 Video S2

Confocal timelapse of caged RNA droplet (DrA_{HHRz}), dissolving upon addition of the HHRz. Scale bar: 50 µm. Droplet contains MGA and is labeled with malachite green dye, $\lambda_{ex} = 640\,\text{nm}$, fluorescence and brightfield overlay. From the starting point up to 47 min, every frame is separated by a 30 s time interval. Timestamps are shown on the top left.

4.3 Video S3

Confocal timelapse of caged RNA droplet (DrA_{HPRz}), dissolving upon addition of HPRz. Scale bar: 50 µm. Droplet contains MGA and is labeled with malachite green dye, $\lambda_{ex} = 640\,\text{nm}$, fluorescence and brightfield overlay. From the starting point up to 16 h, every frame is separated by a 15 min time interval. Timestamps are shown on the top left.

4.4 Video S4

Confocal timelapse of caged RNA droplet (DrA_{HPRz}), first growing and then shrinking upon addition of the IVT mixture containing the DNA template for the HPRz, transcribing the ribozyme *in situ*. Growth of the RNA droplet is due to transcription of new droplet material alongside the HPRz. Scale bars: 50 µm. Droplet contains MGA and is labeled with malachite green dye, $\lambda_{ex} = 640\,\text{nm}$, fluorescence and brightfield overlay. From the starting point up to 16 h, every frame is separated by a 15 min time interval. Timestamps are shown on the top left.

4.5 Video S5

Confocal timelapse of caged RNA droplet, additional replicate of (DrA_{HPRz}), first growing and then shrinking upon addition of the IVT mixture containing the DNA template for the HPRz, transcribing the ribozyme *in situ*. Growth of the RNA droplet is due to transcription of new droplet material alongside the HPRz. Scale bars: 50 µm. Droplet contains MGA and is labeled with malachite green dye, $\lambda_{ex} = 640\,\text{nm}$, fluorescence and brightfield overlay. From the starting

point up to 16 h, every frame is separated by a 15 min time interval. Timestamps are shown on the top left.

4.6 Video S6

Confocal timelapse of mixed, caged RNA droplets (DrA, DrB, L_{HHRz}) segregating upon addition of the transcribed HHRz. Red droplets contain malachite green aptamer (MGA) and are labeled with malachite green dye, $\lambda_{ex} = 640$ nm, blue droplets contain BrA and are labeled with DFHBI-1T, $\lambda_{ex} = 488$ nm, droplets are mixed in the beginning using a ratio of 1:2:1 (DrA:L:DrB). From the starting point up to 14 min 15 s, every frame is separated by a 45 s time interval followed by a short 80 s pause to seal the chamber. Imaging then continued until 1 h 4 min 5 s, with a 45 s time interval, after which the interval was extended to 10 min for overnight acquisition. Timestamps are shown on the top left.

References

1. Fabrini, G. *et al.* Co-transcriptional production of programmable RNA condensates and synthetic organelles. *Nature Nanotechnology,* 1–9 (2024).
2. Conrad, T., Plumbom, I., Alcobendas, M., Vidal, R. & Sauer, S. Maximizing transcription of nucleic acids with efficient T7 promoters. *Communications Biology* **3,** 439 (2020).
3. Geary, C., Grossi, G., McRae, E. K., Rothemund, P. W. & Andersen, E. S. RNA origami design tools enable cotranscriptional folding of kilobase-sized nanoscaffolds. *Nature chemistry* **13,** 549–558 (2021).
4. Fornace, M. E. *et al.* NUPACK: analysis and design of nucleic acid structures, devices, and systems (2022).
5. Hofacker, I. L. Vienna RNA secondary structure server. *Nucleic acids research* **31,** 3429–3431 (2003).
6. Möller, S., Weisser, J., Bischoff, S. & Schnabelrauch, M. Dextran and hyaluronan methacrylate based hydrogels as matrices for soft tissue reconstruction. *Biomolecular Engineering* **24,** 496–504 (2007).
7. Schindelin, J. *et al.* Fiji: an open-source platform for biological-image analysis. *Nature methods* **9,** 676–682 (2012).
8. Rueden, C. T. *et al.* ImageJ2: ImageJ for the next generation of scientific image data. *BMC bioinformatics* **18,** 1–26 (2017).
9. Cerritelli, S. M. & Crouch, R. J. Ribonuclease H: the enzymes in eukaryotes. *The FEBS journal* **276,** 1494–1505 (2009).

5 Supplementary data

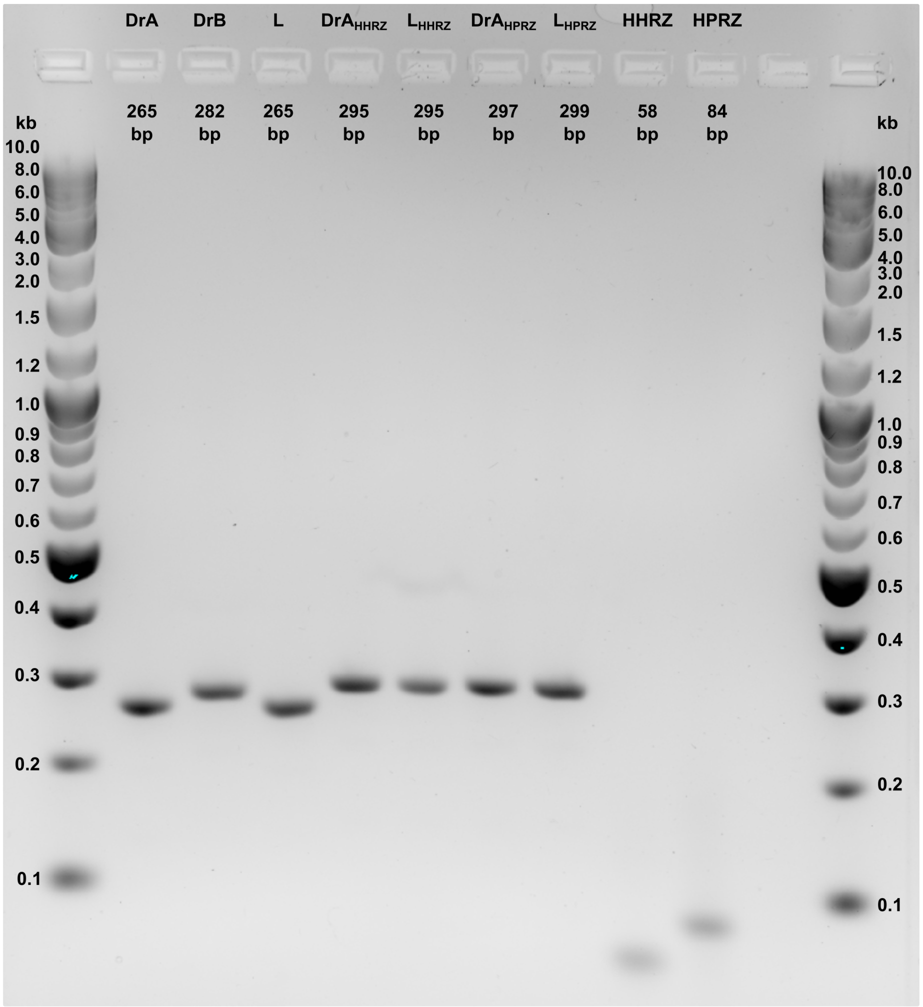

Figure SD1: Agarose gel electrophoresis of PCR products. 3% agarose gel was run at 90 V and stained with Midori Green Advance DNA Stain. TriDye® 1 kb Plus DNA Ladder is used as reference, length in kilo base pairs is reported as text close to the corresponding bands. Visual inspection of the gel confirms the expected sizes of the DNA templates (expected length in base pairs (bp) is reported on top).

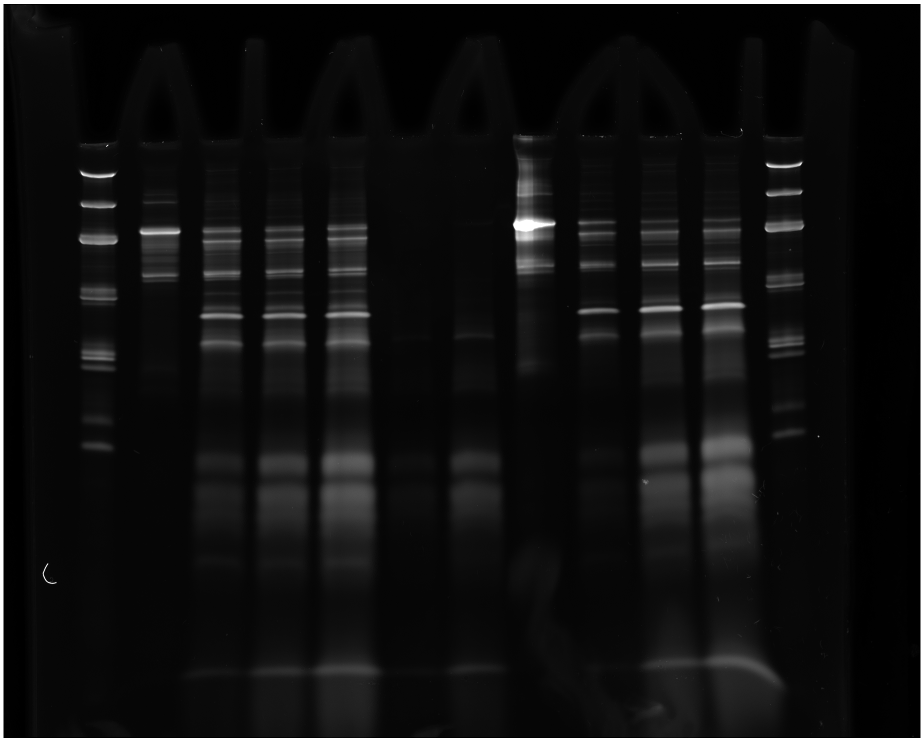

Figure SD2: Uncropped and non-inverted PAGE of cleavage products. Cleavage using the HHRz, corresponding Figure S2.

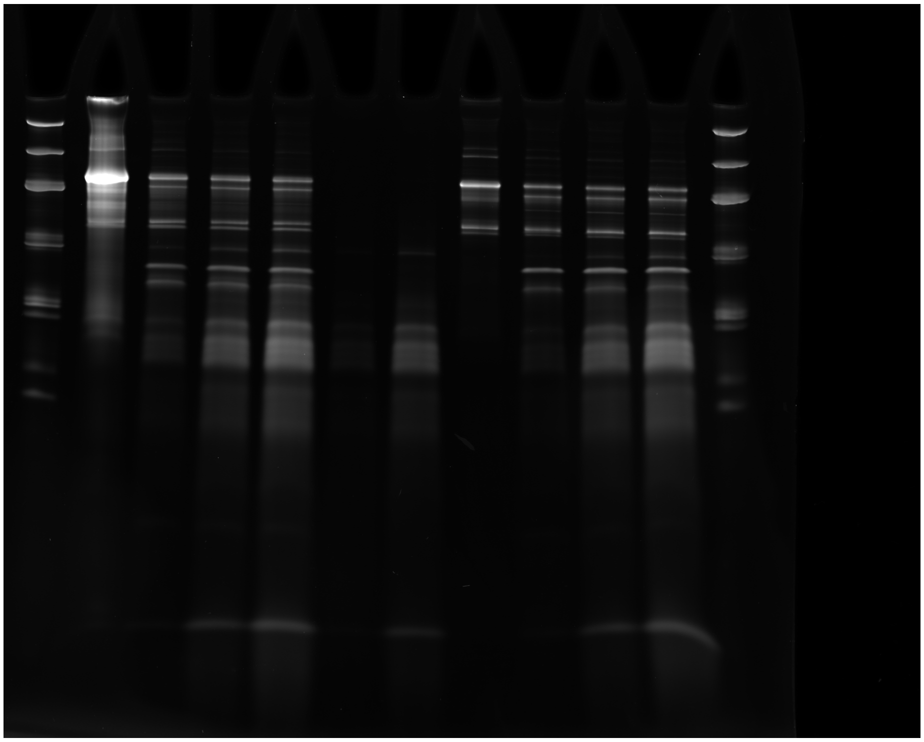

Figure SD3: Uncropped and non-inverted PAGE of cleavage products. Cleavage using the HPRz, corresponding Figure S7.

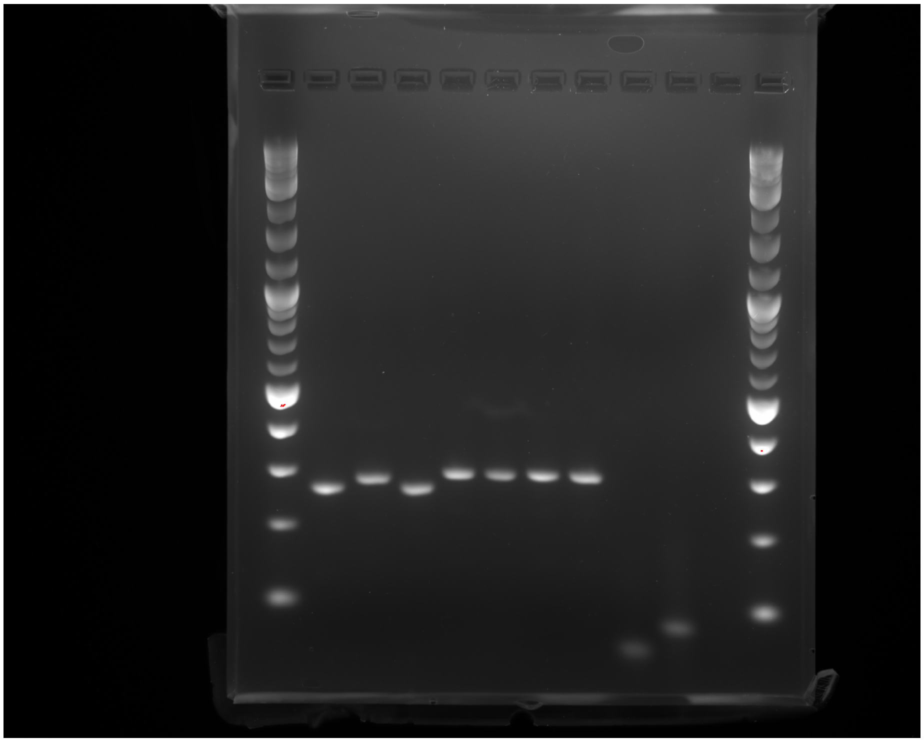

Figure SD4: Uncropped and non-inverted agarose gel electrophoresis of PCR products, corresponding Figure SD1
